# Relationships between Mitochondrial Function, AMPK, and TORC1 Signaling in Lymphoblasts with Premutation Alleles of the FMR1 Gene

**DOI:** 10.3390/ijms221910393

**Published:** 2021-09-27

**Authors:** Paul R. Fisher, Claire Y. Allan, Oana Sanislav, Anna Atkinson, Kevin R. W. Ngoei, Bruce E. Kemp, Elsdon Storey, Danuta Z. Loesch, Sarah J. Annesley

**Affiliations:** 1 Department of Physiology Anatomy and Microbiology, La Trobe University, Bundoora, VIC 3086, Australia; Claire.Allan@latrobe.edu.au (C.Y.A.); O.Sanislav@latrobe.edu.au (O.S.); S.Annesley@latrobe.edu.au (S.J.A.); 2 School of Psychology and Public Health, La Trobe University, Bundoora, VIC 3086, Australia; A.Atkinson@latrobe.edu.au (A.A.); d.loesch@latrobe.edu.au (D.Z.L.); 3 St. Vincent’s Institute of Medical Research, Department of Medicine, University of Melbourne, Fitzroy, VIC 3065, Australia; kevinngoei@gmail.com (K.R.W.N.); Bruce.Kemp@acu.edu.au (B.E.K.); 4 Mary MacKillop Institute for Health Research, Australian Catholic University, Melbourne, VIC 3000, Australia; 5 Department of Medicine, Alfred Hospital Campus, Monash University, Commercial Road, Melbourne, VIC 3004, Australia; elsdon.storey@monash.edu

**Keywords:** AMPK, TOR complex I, mitochondria, FMR1, fragile X-associated tremor/ataxia syndrome (FXTAS), CGG repeat

## Abstract

The X-linked *FMR1* gene contains a non-coding trinucleotide repeat in its 5’ region that, in normal, healthy individuals contains 20–44 copies. Large expansions of this region (>200 copies) cause fragile X syndrome (FXS), but expansions of 55–199 copies (referred to as premutation alleles) predispose carriers to a neurodegenerative disease called fragile X-associated tremor/ataxia syndrome (FXTAS). The cytopathological mechanisms underlying FXTAS are poorly understood, but abnormalities in mitochondrial function are believed to play a role. We previously reported that lymphoblastoid cell lines (LCLs, or lymphoblasts) of premutation carriers have elevated mitochondrial respiratory activities. In the carriers, especially those not clinically affected with FXTAS, AMP-activated protein kinase (AMPK) activity was shown to be elevated. In the FXTAS patients, however, it was negatively correlated with brain white matter lesions, suggesting a protective role in the molecular mechanisms. Here, we report an enlarged and extended study of mitochondrial function and associated cellular stress-signaling pathways in lymphoblasts isolated from male and female premutation carriers, regardless of their clinical status, and healthy controls. The results confirmed the elevation of AMPK and mitochondrial respiratory activities and reduction in reactive O_2_ species (ROS) levels in premutation cells and revealed for the first time that target of rapamycin complex I (TORC1) activities are reduced. Extensive correlation, multiple regression, and principal components analysis revealed the best fitting statistical explanations of these changes in terms of the other variables measured. These suggested which variables might be the most “proximal” regulators of the others in the extensive network of known causal interactions amongst the measured parameters of mitochondrial function and cellular stress signaling. In the resulting model, the premutation alleles activate AMPK and inhibit both TORC1 and ROS production, the reduced TORC1 activity contributes to activation of AMPK and of nonmitochondrial metabolism, and the higher AMPK activity results in elevated catabolic metabolism, mitochondrial respiration, and ATP steady state levels. In addition, the results suggest a separate CGG repeat number-dependent elevation of TORC1 activity that is insufficient to overcome the inhibition of TORC1 in premutation cells but may presage the previously reported activation of TORC1 in FXS cells.

## 1. Introduction

First described in 2001 [1], fragile X-associated tremor/ataxia syndrome (FXTAS) is a late onset, progressive, neurodegenerative disorder characterized by kinetic tremor, gait ataxia, and cognitive decline [2,3]. These major clinical features are accompanied by white matter lesions in the brain, whose most specific locations are in the middle cerebellar peduncles (MCP sign) and the splenium of corpus callosum. The minor features include parkinsonism, neuropathy, or white matter lesions in other brain locations. The term “FXTAS spectrum” has also been used to reflect the large diversity, as well as age-dependence of clinical manifestations of this syndrome [4,5]. 

FXTAS is caused by expansions of the CGG repeats in the 5’ upstream region of the *FMR1* gene from the normal range (<41 repeats, wild type alleles) to the premutation range (55 to 200 CGGs). Rare individuals carrying the alleles of 41–54 CGG repeats (termed “intermediate size” or “gray zone”, GZ, alleles) were also reported to manifest FXTAS, or individual features reminiscent of FXTAS [6]. Not all individuals carrying the premutation (PM) alleles exhibit the clinical phenotype of FXTAS; nearly half these carriers do not develop this condition, but some proportion of these non-FXTAS individuals may manifest either its single features, such as kinetic tremor or cognitive decline, or have other health conditions, such as fibromyalgia, seizures, migraine, anxiety/depression, or hypertension, some apparently occurring at higher frequencies than in the general population [7,8,9]. Because *FMR1* resides on the X chromosome, neurological symptoms are typically milder in females, though nearly 30% of female premutation carriers present with early menopause (fragile X-associated primary ovarian insufficiency). A minority of PM carriers remain asymptomatic independent of their age and males with ≤70 repeats have been reported to be at extremely low risk of developing the condition [10].

As with many other neurodegenerative diseases, mitochondrial dysfunction has been implicated in the cytopathology of FXTAS. Several authors have investigated the status of mitochondrial expression and function in post-mortem brains [11] as well as in a range of cultured cells from PM individuals, including fibroblasts [11,12] and peripheral blood lymphocytes [13], finding reduced expression and function of mitochondrial proteins. Our data based on EBV-transformed B lymphocytes (hereafter termed lymphoblasts) derived from individuals carrying PM alleles [5,14] showed that mitochondria in PM lymphoblasts are hyperactive, with elevated but functionally normal activities of all mitochondrial respiratory complexes. Our purpose here is to evaluate this mitochondrial hyperactivity in an expanded study and to investigate its relationships with the size of the CGG expansion and other associated biochemical differences between PM and healthy control lymphoblasts.

Mitochondrial biogenesis and activity in cells are regulated by intracellular stress-sensing and signaling pathways that ensure the mitochondria respond appropriately to varying energy demands from the cell. Two of the key proteins involved in these homeostatic pathways are AMP-activated protein kinase (AMPK), the major sensor of inadequate cellular energy and other stresses, and target of rapamycin complex I (TORC1), which is regulated by signals from AMPK, amino acids, and growth factors [15,16]. Mitochondrial biogenesis and activity are stimulated both by AMPK and TORC1, so the mitochondrial hyperactivity we previously observed in lymphoblasts from a small sample of PM individuals [5,14] could be a response to elevated activity of either or both protein kinases. We previously reported that AMPK activity is elevated in lymphoblasts from PM individuals [5], but there have been no studies of TORC1 activity levels in cells carrying PM alleles. 

Here, we confirm that mitochondrial respiration and AMPK activities are elevated in PM lymphoblasts. TORC1 activities, by contrast, are affected in PM lymphoblasts (regardless of FXTAS status) in two ways—a reduction compared to control cells, combined with a separate, positive correlation with expansion size within the PM group. Surprisingly, but consistent with our previously reported findings [14], these processes are accompanied not by elevation but by reduction in the levels of reactive O_2_ species, suggesting a reductive rather than an oxidative stress in these cells.

## 2. Results

### 2.1. Participant Cohorts

The two genetic groups (33 controls and 41 FMR1 premutation carriers) bore alleles of the CGG trinucleotide repeat in the FMR1 locus that were either in the normal size range (20–40, control group), or in the premutation range (55–199, PM group). Given that the determination of CGG repeat numbers is accurate to within one or two repeats, we also included two individuals with expansion sizes of 54 in the PM group, bringing the total to 43. Female participants were included in the study, using the CGG expansion size of the larger FMR1 allele, as previous studies had shown that expression of FMR1 mRNA is correlated with this in females, as is the case for the single allele in males [17]. Both age and gender were included as potentially significant variables in the analyses.

#### 2.1.1. Participant Age and Gender Distribution

**Figure 1:**
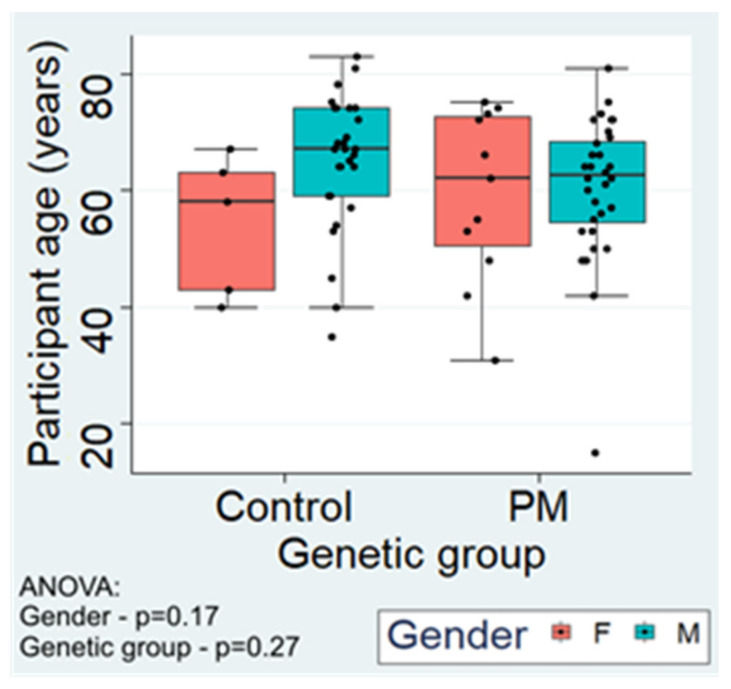
Participant ages in genetic and gender groups. The age distribution of participants grouped by genotype and gender was similar. The indicated *p* values are from analysis of variance for effects of gender (F = female, M = male) and genetic group (Control, PM = premutation). Similar values were obtained from pairwise two-sample comparisons using the nonparametric Mann–Whitney U test (gender—*p* = 0.21, genetic group—*p* = 0.20). The proportions of males and females in the two genetic groups were not significantly different (McNemar’s χ^2^ test, *p* = 0.27; Fisher’s 2-sided exact test, *p* = 0.40) with more males in both groups (85% control, 75% premutation). For the six repeat participants, only information from the 2nd sampling is included.

The maximum ages were in the early 80s in both groups (83 in the control group and 81 in the premutation group) with all but two participants (a 31 year old, unaffected PM female and an autistic 15 year old PM male) being aged 35 or older. There were no significant differences in the age or gender distribution of the two genetic groups (Figure 1).

#### 2.1.2. Clinical Phenotypes and CGG Expansion Sizes

**Figure 2:**
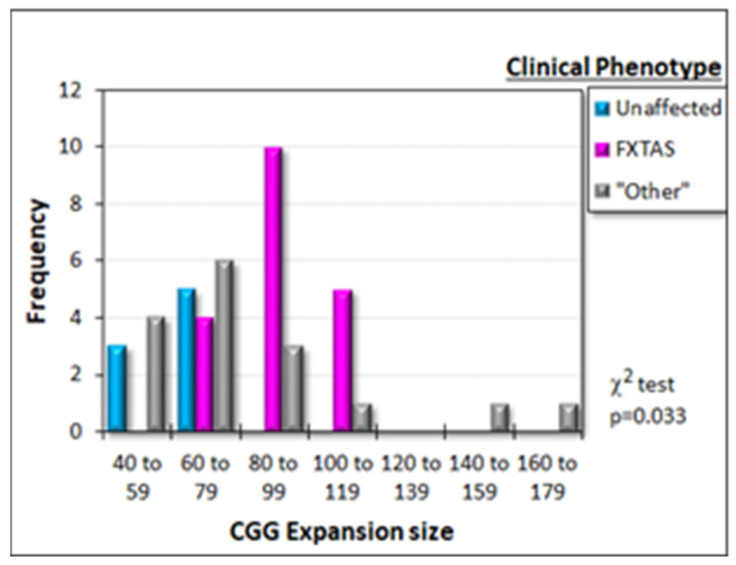
Distribution of clinical phenotypes across the CGG expansion size range in premutation carriers. Most FXTAS participants had expansion sizes greater than 80, while most unaffected PM carriers had expansion sizes less than 80. Other clinical phenotypes were broadly distributed across the whole range of CGG repeat numbers. The χ^2^ test (indicated *p* value) confirmed that these differences amongst the frequency distributions for the three PM carrier groups were statistically significant.

Most symptomatic PM participants exhibited clinically diagnosed FXTAS (19 individuals), while eight individuals were asymptomatic, and 16 individuals manifested isolated features including autism, depression and anxiety, fibromyalgia, imbalance, or orthostatic or kinetic tremor. For the purposes of describing the broad clinical phenotypes of the cohort used, these other clinical presentations are grouped into the “Other” category (Figure 2). As previously reported [5], the number of trinucleotide repeats in the PM carriers differed significantly amongst clinical subgroups (Figure 2). The “Unaffected” group clustered at the low end of the premutation CGG expansion size range, while the “Other” PM carriers mostly had expansion sizes less than 80, but the distribution exhibited a long tail towards larger CGG sizes. Individuals in the “FXTAS” group exhibited CGG expansion sizes with an apparent peak in the 80–99 repeat size range (Figure 2). Only three individuals (all males) in the “FXTAS” group had CGG repeat numbers ≤70, consistent with the relative rarity of FXTAS at the low end of the PM expansion size range [5,10]. The cohort of premutation participants thus exhibited the typically expected range of clinical phenotypes. Elsewhere, we explore the relationships between these phenotypes and the biochemical variables [18], but our purpose in this paper is to investigate potentially causal interrelationships amongst the molecular abnormalities found in premutation lymphoblasts compared to healthy control cells.

### 2.2. Mitochondrial Function, ATP Steady State Levels, Redox Balance and Associated Signaling Activities Are Abnormal in PM Lymphoblasts

**Figure 3:**
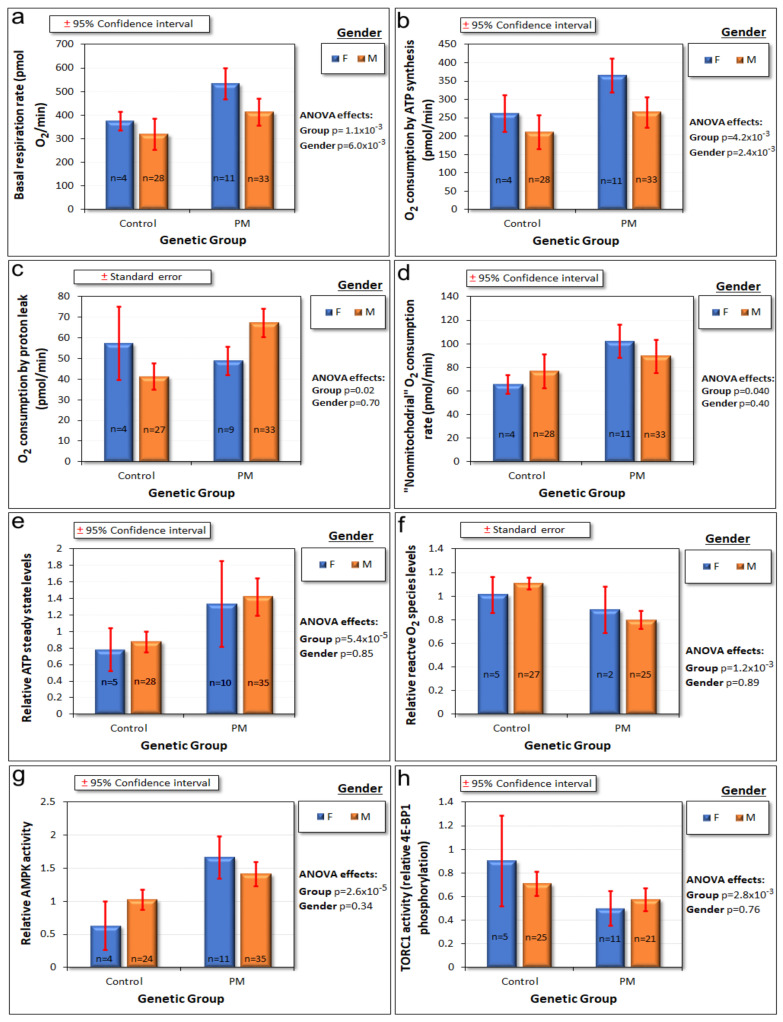
Respiration rates, reactive O_2_ species (ROS) and ATP steady state levels, and stress kinase signaling activities are abnormal in lymphoblasts carrying premutation alleles. A variety of molecular and cellular parameters associated with mitochondrial function were assayed. Two-way analysis of variance showed that many of the measures were either elevated or reduced in PM lymphoblasts (panels (**a**–**h**)), while several were not significantly changed (Supplementary Figure S1). Basal mitochondrial respiration rates and O_2_ consumption attributable to ATP synthesis were also affected by gender (panels (**a**,**b**)).

We previously reported significant elevation of mitochondrial respiratory function [14] in lymphoblasts from a small sample of 16 male PM carriers. In this enlarged study, we confirmed these findings, showing elevated mitochondrial activity in PM lymphoblasts, including the O_2_ consumption rate (OCR) by basal respiration and its components (Figure 3a–d). In cases where there are deficiencies in oxidative phosphorylation, it is possible for the relative contributions of these individual components of basal respiration to change. However, there were no significant differences in the *fraction* of basal respiration attributable to ATP synthesis (*t* test, *p* = 0.67), the “proton leak” (*t* test, *p* = 0.16) or “nonmitochondrial” respiration (*t* test, *p* = 0.15). Similarly, there was no significant difference in the *fraction* of the maximum uncoupled respiration attributable to Complex I (*t* test, *p* = 0.11). This means that all the individual components of respiration in the PM cells are functionally normal, each contributing fractionally to the same extent as in control cells to basal as well as to CCCP-uncoupled respiration. Furthermore, the mitochondrial “mass”, membrane potential and genome copy numbers were the same in the combined sample of PM carriers and control cells (Supplementary Figure S1).

Steady state cellular ATP levels, a product of the balance between mitochondrial ATP synthesis and cellular ATP consumption, were also elevated (Figure 3e). Contrary to reports of elevated oxidative stress in other PM cell types [19,20], the levels of reactive O_2_ species were reduced in the PM lymphoblasts (Figure 3f). Confirming our previous findings [5], the PM lymphoblasts also showed elevated activity of AMPK, the major cellular energy stress-sensing protein kinase, which upregulates mitochondrial biogenesis and activity (Figure 3g). However, the PM cells had reduced activity of target of rapamycin complex I (TORC1), another protein kinase that plays a central role in cellular stress sensing and regulation of mitochondrial biogenesis and respiratory activity (Figure 3h). TORC1 is inhibited both directly and indirectly by AMPK [15,16]. These findings of abnormalities in PM lymphoblasts in the levels of mitochondrial and associated functions prompted us to investigate the relationships amongst them and the underlying genetic cause, the larger than normal CGG repeat numbers in PM cells.

Individual components of mitochondrial respiration in control and PM lymphoblasts are strongly correlated with each other and less strongly with steady state ATP levels in PM cells.

As a first step in teasing out the most significant relationships amongst the abnormal biochemical properties of PM lymphoblasts, we examined the correlations amongst the individual components of respiration and its immediate product, cellular ATP. Basal respiration in cells has three components measurable in our assays—O_2_ consumption rates (OCR) attributable to ATP synthesis by Complex V, to other mitochondrial membrane transport processes (“proton leak”) and to direct use of O_2_ by enzymes that are ***not*** involved in mitochondrial oxidative phosphorylation (“nonmitochondrial” O_2_ consumption). We also measured the maximum OCR after using the protonophore CCCP to abolish the proton gradient, thereby uncoupling electron transport from ATP synthesis. Complex I activity was determined as the decrease in the uncoupled OCR after addition of rotenone, a Complex I inhibitor. The use of the proton gradient for other mitochondrial membrane transport processes (the “proton leak”) was calculated as the difference between the oligomycin-inhibited OCR and the nonmitochondrial OCR. All these linked respirometric measures of mitochondrial function were highly positively correlated with each other (Table 1).

Unlike the other Seahorse respirometry measures, the “nonmitochondrial” component of basal respiration is not directly connected mechanistically to oxidative phosphorylation. Instead, it measures the combined activity of other O_2_-consuming hydroxylases and oxygenases in the cell. It may thus be regarded as a surrogate measure of the rate of nonmitochondrial metabolism in the cell. The results in Table 1 show that in both control and PM cells, the “nonmitochondrial” OCR was strongly correlated with the mitochondrial components of cellular O_2_ consumption. This is what is expected if, in both control and PM lymphoblasts, the production of ATP in the mitochondria is regulated homeostatically to meet the metabolic demand.

Although cellular demand for, and generation of, ATP are balanced at steady state, the cellular ATP levels at which this is achieved can differ, depending on the physiological state of the cell. Table 1 shows that, in control lymphoblasts, steady state ATP levels were not correlated with ATP synthesis rates or any of the other measured components of respiration. By contrast, in the PM cells, the steady state ATP levels (at which ATP production and consumption are balanced) were significantly correlated with basal and maximum OCR and their major components (ATP synthesis and Complex I activity, respectively). These results suggest that the CGG expansions in cells with premutation alleles cause them to reside in an altered physiological and regulatory state in which ATP production and consumption become balanced at higher steady state ATP levels.

### 2.3. Multiple Regression Relationships between CGG Repeat Number, Mitochondrial Activity, and Cellular Stress Signaling by AMPK and TORC1

The mechanisms by which cells monitor their energy needs and regulate mitochondrial biogenesis and activity to match demand are known to involve a network of cellular stress-sensing pathways. This being the case, we used correlation and multiple regression analysis to investigate the interrelationships between premutation alleles and multiple parameters of mitochondrial activity and their potential regulators. We used dummy variables to allow both the slopes and intercepts of regression lines to differ between the control and PM groups so that we could also detect possible differences in the regulatory relationships in lymphoblasts in the two genetic groups. We also included participant gender and age in the analyses to reveal their potential modifying effects. When, as here, there are multiple correlated variables, multiple regression analysis can reveal which are the most important variables in statistically “explaining” a dependent variable. As with all relationships amongst variables measured in clinical studies, this cannot demonstrate causal connections, but it can provide indications as to which of the tested explanatory variables might be the most proximal (i.e., the closest in a chain or network of causal links) regulators of a given cellular activity.

#### 2.3.1. Elevated AMPK Activity Can Explain the Elevated Rates of Mitochondrial Oxidative Phosphorylation in PM Lymphoblasts

**Figure 4:**
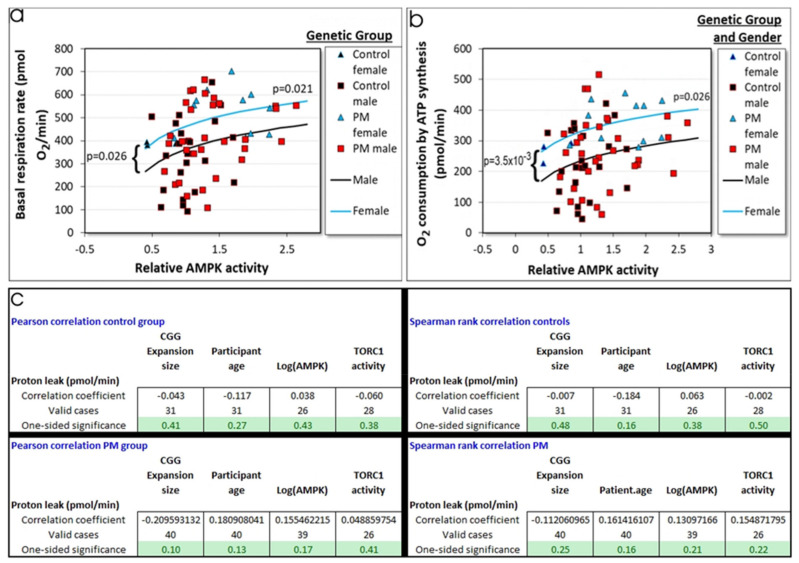
Mitochondrial oxidative phosphorylation is related to AMPK activity levels in PM and control lymphoblasts. Multiple regression analysis was conducted of basal respiration (**a**) and its two measured mitochondrial components, ATP synthesis (**b**) and the “proton leak” (**c**). The explanatory variables were the patient age and gender, logarithms of CGG repeat number, AMPK and TORC1 activities, as well as dummy variables allowing relationships for the two genetic groups (control and PM) to differ in slope and/or intercept. After stepwise addition of significant and removal of insignificant variables, only the regression relationships between the oxidative phosphorylation rate and AMPK activity in the cells were significant (*p* values as indicated, complete details of the multiple regression analysis in Supplementary Regressions 1 and 2). These regression relationships with AMPK activity were confirmed by robust regression for both basal respiration (*p* = 3.2 × 10^−3^) and ATP synthesis rates (*p* = 7.1 × 10^−3^). In both cases, there was a significant constant additional effect of gender (cyan lines), with respiration rates being higher in lymphoblasts from female participants. This result was confirmed by robust regression for both basal respiration (*p* = 7.2 × 10^−3^) and ATP synthesis (*p* = 7.6 × 10^−5^). The control cell lines tended to cluster at lower and the PM cell lines at higher respiration rates and AMPK activities. The “proton leak” showed no significant regression relationship in standard (Supplementary Regression 3) or robust regression and no significant Pearson or Spearman correlation with any of the variables tested, as confirmed by parametric and nonparametric correlation analysis within each genetic group (**c**).

Mitochondrial hyperactivity in PM lymphoblasts could be regulated by AMPK or TORC1 or indirectly (via other pathways) by the large CGG expansions. We examined the relationships between the basal respiration rate (Figure 4a), as well as O_2_ consumption by its two mitochondrial components—ATP synthesis (Figure 4b) and the “proton leak” (Figure 4c) using the genetic group, CGG repeat number, AMPK activity, TORC1 activity, participant age, and gender as explanatory variables. The multiple regressions included dummy variables allowing the intercepts and slopes of the regression relationships to differ between the groups. After stepwise removal of insignificant variables, the final regression models for basal respiration and O_2_ consumption by ATP synthesis included significant terms for an intercept constant, an additional constant effect of gender and a nonzero slope for the relationship between AMPK activity and the basal respiration and ATP synthesis rates in the cells. This result was confirmed in lasso regression analysis (Supplementary lasso regressions 1 and 2). Thus, once the participant gender and AMPK activity were taken into account, there was no additional significant difference between the PM and control groups and no additional effect of CGG repeat number or TORC1 activity.

In ANOVA (Figure 3c) and two sample tests (*p* = 0.02), the “proton leak” was also significantly elevated in the PM cells compared to controls. However, this difference could not be accounted for by a statistically significant relationship in multiple regressions or correlation analysis (Figure 4c) or lasso regressions (Supplementary Lasso Regression 3) with any one of the potential explanatory variables tested—patient age or gender, CGG repeat number, AMPK activity, or TORC1 activity. This is likely to be due to the relatively high noise to signal ratios for proton leak measurements that result from the relatively small contribution of the proton leak to basal respiration (standard deviation equals ca. 70% of the mean in the Seahorse respirometry assays) (Figure 3c). Given the strong correlation between the proton leak and basal respiration, it seems likely that proton leak is also AMPK-dependent, but this needs to be tested in larger samples.

We conclude that, of the explanatory variables tested, the elevated AMPK activity is sufficient to explain the elevated rates of mitochondrial oxidative phosphorylation in both the male and female PM cells, with the female cells in our small sample exhibiting a further significant elevation that is AMPK-independent. This result favors a model in which AMPK is the most proximal activator of mitochondrial function in both the PM and control cells.

**Figure 5:**
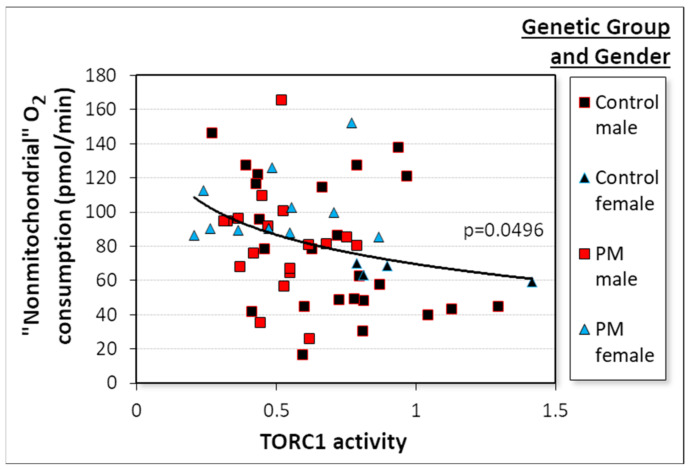
Nonmitochondrial respiration is negatively related to TORC1 activity. Multiple linear regression of the “nonmitochondrial” component of basal respiration using as explanatory variables the CGG repeat number, patient age and gender, ATP steady state levels, AMPK, and TORC1 activity. The significance of the final regression model was as indicated. The result was confirmed in robust regression (*p* = 6.1 × 10^−3^). Control cell lines tended to cluster at higher TORC1 and lower “nonmitochondrial” O_2_ consumption rates, while PM cell lines did the reverse.

The final component of basal respiration that was elevated in the PM cells was the “nonmitochondrial” O_2_ consumption. In this case, the multiple log–linear regression analysis showed that after stepwise removal of insignificant variables; this component of respiration was negatively related to the activity of TORC1 (Figure 5, Supplementary Regression 4, Supplementary Lasso Regression 4). Once this was accounted for, the other potential explanatory variables had no significant additional effect. This result supports the possibility that TORC1 is the most proximal regulator of the nonmitochondrial component of respiration. Its reduced activity in the PM cells (Figure 3h) could thus be responsible for the increased rate of “nonmitochondrial” O_2_ consumption (Figure 3d) in these cells.

#### 2.3.2. AMPK Activity in Control and PM Lymphoblasts Depends on TORC1 Activity, Which Depends on the CGG Repeat Number

The results presented thus far revealed no differences between control and PM cells in the functional relationships between AMPK, TORC1, and respiration rates in control and PM lymphoblasts. The elevated mitochondrial and “nonmitochondrial” components of respiration could be statistically accounted for by the higher AMPK activity and lower TORC1 activity in the PM cells. This raises the question as to how these two activities relate to each other and to the CGG repeat numbers. In other cell types, AMPK and TORC1 exhibit mutually inhibitory actions, with AMPK inhibiting TORC1 directly (by phosphorylating raptor) and indirectly (via phosphorylation of Tsc1), while TORC1 feeds back to inhibit AMPK both directly (by phosphorylating a_1_, the catalytic subunit expressed in lymphoid cells at S_347_) and indirectly (via an activating phosphorylation of Tsc2) [15,16,21]. We previously reported that AMPK activity was elevated in PM lymphoblasts [5]. Here, we confirm this result in larger samples, as well as reporting that TORC1 activity is reduced in PM lymphoblasts compared to controls.

**Figure 6:**
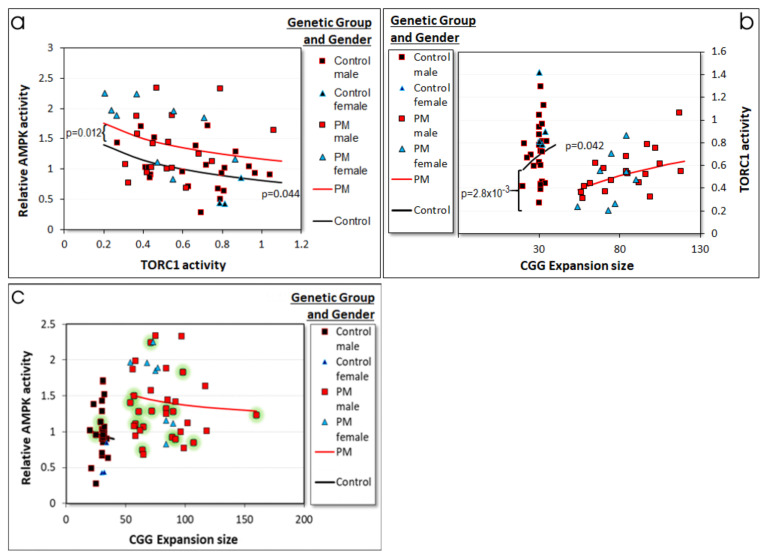
Relationships between CGG repeat number, TORC1, and AMPK activities in control and PM lymphoblasts. (**a**) The AMPK activity was negatively correlated with TORC1 activity in both the control and the PM cells, and the slope of the regression line was the same in both cell types. In addition, the PM cells exhibited a constant TORC1-independent elevation of AMPK activity (difference in intercepts). The lines are those predicted by the multiple regression result and the significances of the regression coefficients were as indicated. Details of the multiple regression are presented in Supplementary Regression 5. The result was confirmed by robust regression, which showed a significant TORC-independent elevation in PM cells (*p* = 0.01), as well as a significant negative relationship with TORC1 activity in both cell types (*p* = 5.5 × 10^−4^). (**b**) There was a clear difference in the intercepts, but not the slopes of the log–linear regressions of TORC1 activity versus CGG repeat number. This means that in the PM and control groups the TORC1 activity increased at the same rate with increasing CGG repeat number in the log–linear regression (slopes identical and significant at the *p* value shown at the center of the chart). However, the extrapolated starting point (the intercept—at a theoretical repeat number of 0) is much lower for the PM group (significance of the difference indicated by the *p* value on the left). This significantly lower intercept caused the overall decrease in TORC1 activity in Figure 3h. The lines are those predicted by the multiple regression result (Supplementary Regression 6). The results were confirmed in robust regressions for both the positive correlation with repeat number (*p* = 7 × 10^−3^) and the difference in intercepts between PM and control cells (*p* = 2.5 × 10^−4^). (**c**) The regression relationships in panels (**a**,**b**) were used to predict the relationship between AMPK activity and CGG repeat number in the two genetic groups (black and red lines). These are plotted on a scatter plot of the actual data, including cell lines (green halos) for which we do not have TORC1 activities, and which, therefore, did not contribute to the determination of the regression relationships.

We performed a regression analysis to investigate the dependence of AMPK activity on the potential explanatory variables, with TORC1 activity, CGG repeat number, and participant age, gender, and genetic group as the potential explanatory variables (Figure 6a, Supplementary Regression 5). After progressive stepwise removal of insignificant variables, the AMPK activity was found to exhibit both an elevation in PM cells compared to controls that was independent of all the explanatory X variables (higher intercept) and to be negatively related to TORC1 activity (in both genetic groups). This result was confirmed by lasso regression (Supplementary Lasso Regression 5). It suggests that two processes regulate AMPK activity in cells with PM alleles, a TORC1-dependent inhibition of AMPK activity both in control and PM cells and a TORC1-independent elevation of AMPK activity found only in PM cells. Once these two effects were accounted for, the other variables provided no additional significant effects (Figure 6a). The negative correlation is consistent with the known mutually inhibitory actions between AMPK and TORC1. The fact that the regression slope was the same in the PM and control cells shows that this mutually inhibitory relationship is functionally unchanged in PM lymphoblasts.

A similar multiple regression analysis using TORC1 activity as the dependent variable revealed a dramatic decrease in the PM group that was unaffected by any of the other variables tested (significantly lower intercept term in the log–linear regression). This was accompanied by a CGG repeat number-dependent increase in activity that was present in both control and PM cells (Figure 6b). Separate correlation analysis of the TORC1 and CGG expansion sizes within each of the genetic groups alone confirmed the positive correlation within the PM group (Pearson *p* = 5.6 × 10^−3^, Spearman *p* = 0.016) but not the control group (Pearson *p* = 0.20, Spearman *p* = 0.24). In lasso regression analysis however, a correlation was not detected in either the control or the PM group (Supplementary Lasso Regression 6). Thus, it remained possible that the additional positive relationship between CGG repeat number and TORC1 activity is actually restricted to the PM group or is not present in either PM or control cells.

To explore this further, we used the regression relationship between AMPK and TORC1 activities, as well as that between TORC1 activity and CGG repeat number to predict the expected relationship between AMPK activity and repeat numbers. This provided an opportunity to verify the robustness of the interrelationships observed between AMPK, TORC1, and CGG repeats. The result is shown in Figure 6c (red and black lines) along with the actual measurements (data points). For technical reasons (death of some cell lines, technical experimental failure), we do not have TORC1 activities for some cell lines. Results for these cell lines therefore did not contribute to determination of the regression relationships in Figure 6a,b, so that the data in Figure 6c effectively includes a training set (TORC1 activity available) and a test set (no TORC1 activity data). The prediction of elevated AMPK activity in PM cells, combined with a shallow negative relationship between it and CGG expansion size within each genetic group fitted the data well, both for the training and test sets. This could be consistent with our earlier finding that the severity of cognitive and motor deficiencies and of brain white matter lesions in FXTAS patients are positively correlated with CGG expansion sizes, but negatively correlated with AMPK activity in lymphoblasts [5].

As another way of determining the true regression relationships between the TORC1 and AMPK activities and CGG repeat numbers, we applied multivariate lasso regression (multiple dependent variables) using TORC1 and AMPK activities as the dependent Y variables and the genetic group and repeat numbers as the independent X variables. This technique allows determination of the relationship between multiple dependent variables and a set of independent variables, while also taking into account the covariance relationships between the dependent Y variables. The results for the best fitting lasso regression confirmed that AMPK activities are elevated and TORC1 activities reduced in PM cells and again identified an additional positive relationship between CGG repeat number and TORC1 activity (Supplementary Lasso Regression 7). This confirmed the results of the ordinary multiple regression analysis. However, the predicted weak, negative relationship between AMPK activity and CGG repeat number *within* each genetic group (Figure 6c) was not supported either by the lasso multivariate regression or by separate, normal multiple regression analysis. This suggests that the AMPK activity is controlled by additional factors and not just the combined effects of the enlarged CGG expansions and reduced TORC1 activity in PM cells.

#### 2.3.3. Elevated ATP Steady State Levels in PM Lymphoblasts Depend on AMPK Activity in PM Cells

**Figure 7:**
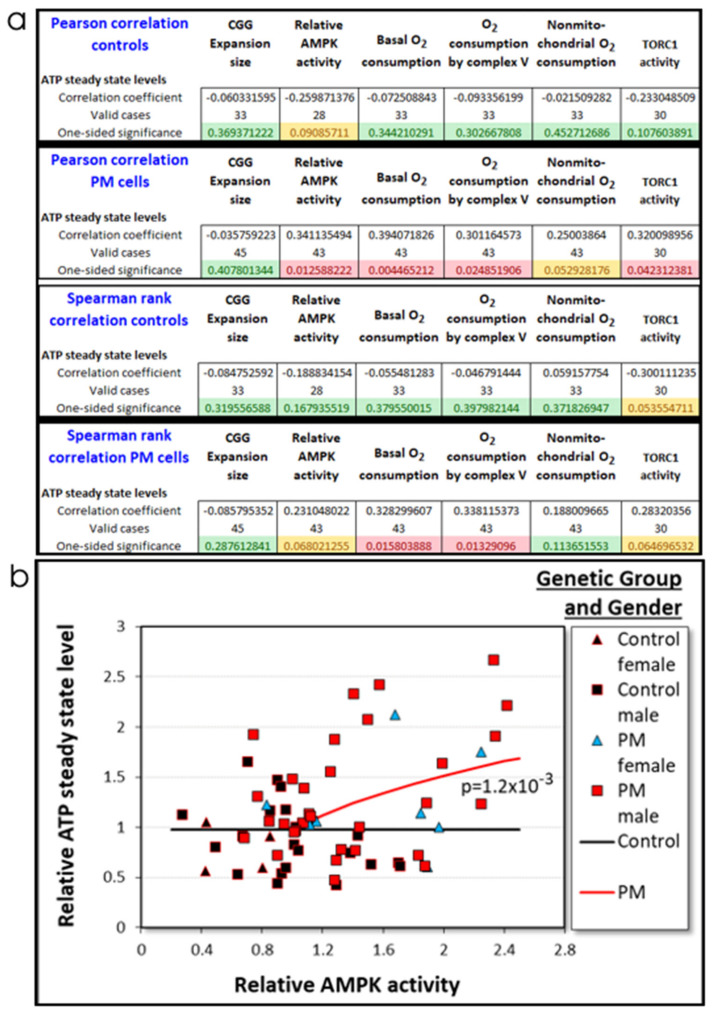
ATP steady state levels depend upon the AMPK activities in PM cells. (**a**) Parametric (Pearson) and nonparametric correlations were determined between steady state ATP levels and possible explanatory variables—CGG repeat number, AMPK and TORC1 activity, as well as basal and ATP synthesis, and nonmitochondrial O_2_ consumption rates. There were no significant correlations in the control cells, but in the PM cells, the steady ATP level was significantly correlated with both kinase activities as well as the OCR by basal respiration and ATP synthesis (Pearson). A similar trend was observed with the Spearman coefficient, except that the correlations with the kinase activities did not reach statistical significance. (**b**) The possible explanatory variables in panel (**a**) were all included in a multiple log–linear regression analysis, along with the participant age, gender, and genetic group, the latter two of which affected the rates of mitochondrial oxidative phosphorylation (Figure 4a,b). After stepwise removal of insignificant variables, the ATP steady state levels in the PM group exhibited a significant relationship with AMPK activities (*p* value indicated, regression details in Supplementary Regression 7).

Table 1 showed that ATP steady state levels were positively correlated with mitochondrial basal respiration, ATP synthesis, maximum respiratory capacity, and complex I activity in the PM cells, but not the control cells. ATP steady state levels are a result of the balance between ATP production and consumption as well as of the regulatory feedback loops that control them. Thus, the respiratory activities that could be the most proximal mediators of ATP steady state levels are the ATP synthesis rates (measured by ATP synthase O_2_ consumption) and ATP consumption rates (using “nonmitochondrial” O_2_ consumption as a surrogate indicator of nonmitochondrial metabolism). The basal metabolic rate includes both of these components as well as the use of the mitochondrial proton gradient for diverse mitochondrial transport processes (the “proton leak”). In addition to these, the proximal controllers of ATP steady state levels could include the regulatory activities of AMPK and TORC1 in controlling catabolic and anabolic processes in the cell. Furthermore, the CGG expansions in PM cells could activate other pathways not tested in this work which then in turn control ATP demand or production. The results in Figure 4a,b had shown that, in our sample, the female PM cells had elevated mitochondrial basal respiration and ATP synthesis rates, so that participant gender could also control nonmitochondrial metabolic rates. We therefore used all these possible regulators as explanatory variables in correlation (Figure 7a) and multiple regression analysis (Figure 7b) of ATP steady state levels. The final regression model after stepwise removal of nonsignificant variables included only the relationship with AMPK activities in the PM cells (Figure 7b) confirmed by Supplementary Lasso Regression 8). This result means that the elevated ATP steady state levels in the PM cells are best explained not by the mitochondrial ATP synthesis rates alone, but also by AMPK’s coordinate inhibition of anabolic ATP-consuming activity and activation of ATP-generating catabolism that provides the mitochondria with oxidizable substrates.

#### 2.3.4. Reactive O_2_ Species (ROS) Levels Are Reduced in PM Lymphoblasts in a CGG Repeat Number-Dependent Manner

**Figure 8:**
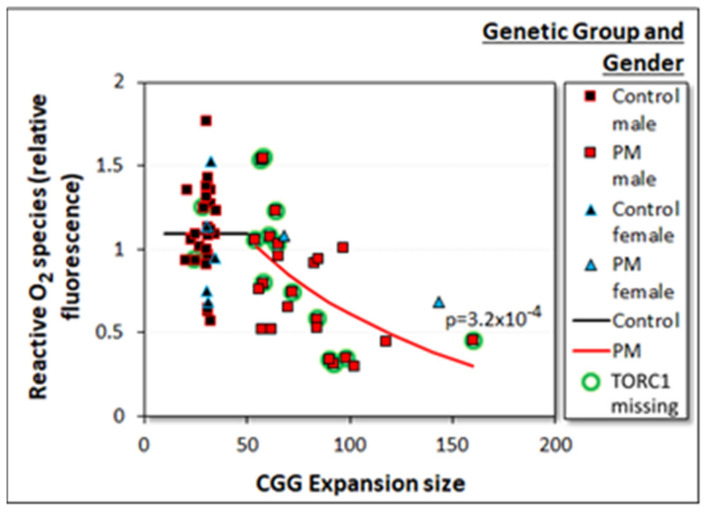
Reactive O_2_ species levels decline with increased CGG repeat number in PM but not control lymphoblasts. Multiple log–linear regression analysis of ROS levels was conducted with CGG repeat number, the activities of AMPK and TORC1 as well as basal respiration rates, gender, and genetic group as the explanatory variables. Dummy variables were included to allow each genetic group to have different intercepts and slopes. After stepwise removal of insignificant variables, only the CGG repeat number in the PM group was significant (red line, *p* value shown). Within the control group (black line), there was no significant relationship between ROS levels and any of the explanatory variables (*X* axis). Complete details of the multiple regression analysis are provided in Supplementary Regression 8. The plot also shows results for cell lines for which TORC1 activities were not available and which therefore did not contribute to the multiple regression analysis (green halos).

FXTAS is a neurodegenerative disease in which it has been proposed that cumulative reactive O_2_ species (ROS) damage may contribute to neuronal death [22]. We therefore also measured ROS levels in the control and PM lymphoblasts. Since ROS production occurs primarily in the electron transport chain, it was possible that the ROS levels might be primarily a function of mitochondrial respiration rates, which were elevated in the PM cells. In confirmation of our previous findings [5], ANOVA showed that ROS levels were lower in the PM group (Figure 3f). Multiple regression confirmed this, showing that the ROS levels in PM but not control cells were negatively correlated with the CGG repeat number (Figure 8), and that once this was accounted for, there was no additional significant effect of basal respiration rates, AMPK activity, or TORC1 activity. Like ATP steady state levels, ROS levels in cells are a result of a balance between two processes—ROS removal by endogenous antioxidants and ROS production by leakage from the electron transport chain, e.g., due to impaired electron flow to O_2_ from complexes I and II via complexes III and IV. The reduced ROS levels in the PM cells are inconsistent with any impairment in mitochondrial electron transport (which would cause elevated ROS levels), supporting the respirometry measurements, which showed that the respiratory complexes in the PM cells are functionally normal (see earlier). In the absence of a blockade of electron transport, the simplest explanation for reduced ROS levels is an increase in the levels of endogenous antioxidants such as mitochondrial glutathione reductase and superoxide dismutase, as well as ROS-trapping metabolites. Such an increase in the form of hyperactivation of the polyol pathway was reported previously by Song et al. [20], leading to elevated levels of ROS traps, such as sorbitol.

### 2.4. Principal Components Analysis Confirms the Major Interrelationships Amongst Measures of Mitochondrial Function and Cellular Stress Signaling in PM Lymphoblasts

**Figure 9:**
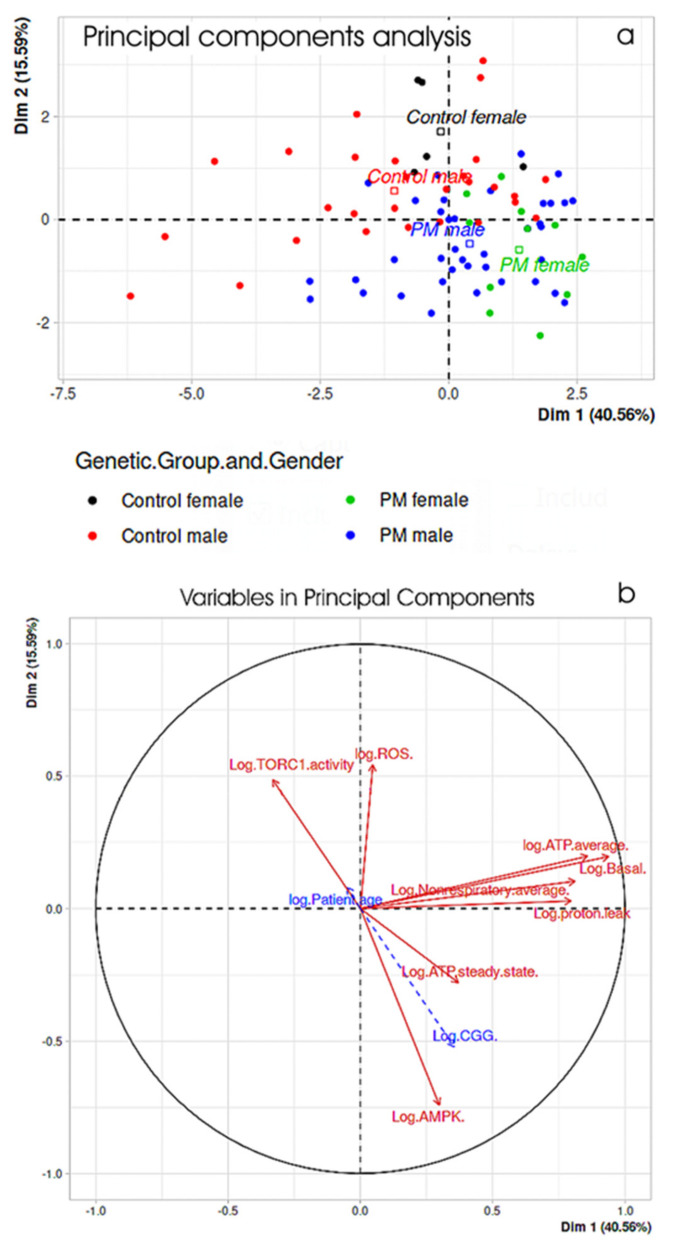
Principal components analysis of cellular stress signaling and mitochondrial function in PM and control lymphoblasts. Basal respiration and its components, as well as ATP steady state and ROS levels, AMPK, and TORC1 activities were all combined in a principal components analysis. (**a**) The first two principal components for each participant (Dim1 and Dim2) are plotted and together accounted for 56% of the total variance amongst the participants. The PM and control groups were clearly separated and centered (medians indicated by square symbols) in the top left and bottom right quadrants, respectively, while the females within each group were centered to the right of the males (i.e., separated primarily along the Dim1 axis). (**b**) The relative contributions of each variable to the first two principal components are shown (solid red arrows). The measures of mitochondrial respiration contributed primarily to Dim1, while the ROS levels contributed primarily to Dim2. ATP steady state, AMPK activity, and TORC1 activity levels made major contributions to both Dim1 and Dim2. Vectors pointing in similar directions are correlated positively with one another, while vectors pointing in opposing directions are correlated negatively with one another. The CGG repeat numbers and patient age (dashed blue arrows) were included in the analysis as supplementary variables that did not contribute to the analysis. The short vector for patient age reflects its low correlation with the other variables and the overall age-matching of the PM and control groups. The CGG repeat number vector points, as expected, into the lower right (PM) quadrant and thus relates positively to the mitochondrial respiration rates, AMPK activities and ATP steady state levels, but has overall negative relationships with TORC1 activities and ROS levels.

The regression and correlation techniques used in the foregoing analyses revealed the strongest statistical relationships between individual “dependent” and multiple “independent” variables, but no single analysis took into account all variables and their interrelationships at the same time. To do so and to examine the relative contributions of the cellular stress signaling and mitochondrial function measurement to the overall physiological differences between PM and control cells, we used all of them together in a principal components analysis (Figure 9). The results confirmed the major conclusions drawn above, revealing a clear separation of PM and control, male and female cells. Within each of the control and PM groups, the medians for the females are positioned to the right of those for the males along the Dim1 axis, due to the significant elevation of respiratory activity in female lymphoblasts compared to male cells. Consistent with their elevated mitochondrial respiration rates, elevated AMPK activities, higher ATP steady state levels, reduced TORC1 activities and ROS levels, the PM cells were located to the right of the control cells along the Dim1 axis and below them on the Dim2 axis.

## 3. Discussion

Many of the clinical phenotypes produced by premutation alleles are neurological or neurodegenerative in origin, but the underlying cytopathology remains poorly understood. We previously reported that mitochondrial respiratory activity and signaling by AMPK, the key cellular energy-sensing protein kinase, is elevated in immortalized lymphocytes (lymphoblasts) from individuals carrying PM alleles [5,14]. A negative correlation between white matter lesions and AMPK activity in premutation carriers suggested that the increased AMPK activity might be part of a cellular compensatory process that provides protection against white matter damage [5]. In this study we measured a collection of molecular parameters related to mitochondrial function in lymphoblasts from enlarged age- and gender-matched groups of controls and PM allele carriers, some with and some without clinical FXTAS. Our results confirmed our previous findings [5,14] that mitochondrial respiration (Figure 3a–d) and AMPK activity (Figure 3g) are both elevated, while reactive O_2_ species levels are lower in lymphoblasts from our total PM sample. They also revealed for the first time that the activity of another key stress-sensing protein kinase, TORC1, is lower in PM cells (Figure 3f,h). Other commonly used indicators of mitochondrial function—the mitochondrial membrane “mass”, membrane potential and genome copy number were not significantly different between PM and control lymphoblasts (Supplementary Figure S1c–f).

**Figure 10:**
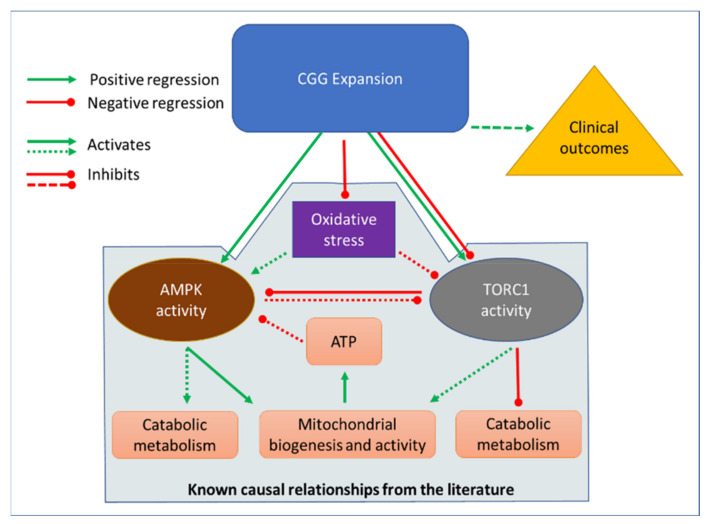
Interactions amongst parameters of mitochondrial function and regulation in PM lymphoblasts. Known network interactions amongst the significantly altered parameters of mitochondrial function are shown. The CGG expansions in PM alleles cause a variety of coupled outcomes in the cells. Solid lines indicate which of these interactions the multiple regression analysis revealed as providing the “best” statistical explanation of the data. These may be considered the most likely candidates for the most proximal causal connections that can explain the observations.

To explore the potentially causal relationships amongst these cellular stress-signaling and mitochondrial activities in cells with enlarged CGG repeats, we used multiple regression and correlation analysis. As shown in the wider context of known causal interactions amongst these parameters (Figure 10), the regression results suggested for each of them the relationship that provided the “best” statistical explanation of the data. These can indicate the most proximal of the known causal relationships in the interaction network, thereby suggesting (but not proving) a potential causal chain of dysregulated cellular and mitochondrial functions. How these molecular and cellular events might in turn relate to the diverse clinical outcomes in carriers of premutation alleles is described elsewhere [18].

In this study, our initial parametric (Pearson) and nonparametric correlation analysis showed that all the components of basal respiration measured using Seahorse respirometry—O_2_ consumption by ATP synthesis, the so-called “proton leak” and the “nonmitochondrial” component of respiration were highly correlated with one another, both in control and PM cells (Table 1, Figure 9). This is expected, as ATP synthesis must be supported by the use of the mitochondrial proton gradient to transport metabolic substrates, products and other molecules across the mitochondrial membrane (the “proton leak”), while it must also match the cellular metabolic demand for ATP, a surrogate indicator of which is the nonmitochondrial O_2_ consumption. Two further respirometric measures of mitochondrial function—the maximum capacity for O_2_ consumption by electron transport and its major component, uncoupled complex I activity also trended upwards in the PM cells, but the elevation did not reach statistical significance (Supplementary Figure S1a,b). The maximum uncoupled respiration rate and complex I activity are a physiological measure of the levels of respiratory electron transport complexes I through IV in the cells, so this result suggests a possible upregulation of the expression of these complexes. Such an upregulation would be expected, given the observed increase in AMPK activity in these cells. In other eukaryotic cells, AMPK upregulates mitochondrial activity in several ways—it phosphorylates the transcriptional cofactor PGC-1α to increase the transcription of genes encoding mitochondrial proteins, it stimulates translocation of glucose transporter proteins to the cell membrane, and it activates catabolism while inhibiting biosynthetic pathways by phosphorylating key rate-controlling enzymes [23]. 

However, AMPK is not alone in regulating mitochondrial function. TORC1 also activates mitochondrial protein expression at the translational level by phosphorylating 4EBP-1 and S6K [24], as well as indirectly at the transcriptional level via YY1 and PGC-1α [25]. AMPK and TORC1 activities are also coupled in mutually inhibitory relationships that form part of a wider network of stress-sensing signaling pathways regulating many cellular activities, most prominently metabolic rate, mitochondrial function and the balance between ATP supply and demand, catabolism, and anabolism [23,24]. Our multiple regression analysis revealed that the elevated AMPK activity in PM lymphoblasts was sufficient to “explain” the mitochondrial hyperactivity of these cells, with no additional significant effects of the CGG repeat number or TORC1 activity. This result suggests that AMPK activity might be the most proximal activator of mitochondrial hyperactivity in PM cells.

Our participant cohort included a relatively small number of females, and within the constraints of the small sample size, we did not detect additional, significant contributions of gender to most of our regression relationships. The exceptions were the mitochondrial basal respiration and its major component, ATP synthesis. Both of these were found to be elevated uniformly in female cells compared to their male counterparts (significantly different intercepts), but the effect of the AMPK activity (regression slope) within each group was the same (Figure 4a,b). The additional AMPK-independent effect of gender on mitochondrial respiration was also not limited to the PM lymphoblasts. Due to the small female sample, especially in the control group, these conclusions need to be verified in larger participant cohorts. In isolated mitochondria from biopsied muscle cells, it has been shown that the intrinsic metabolic rate, respiratory complex activities, and proton leak are higher in females than males [26]. Similar observations have been made in a variety of human and rodent tissues, but the mechanisms remain unclear [27].

Whereas AMPK was the most likely mediator of elevated mitochondrial oxidative phosphorylation in PM cells, the premutation itself (genetic group) as well as the reduced TORC1 activity emerged as the statistically most important explanatory variables for the elevation of “nonmitochondrial” respiration in PM cells (Figure 5). TORC1 is a central controller of metabolism, inhibiting catabolic pathways, and upregulating anabolic pathways. Thus, it makes sense if the lower TORC1 activity in PM cells were to result in elevated nonmitochondrial O_2_-consuming metabolism.

In view of their importance in regulating mitochondrial and nonmitochondrial respiration, we conducted multiple regression analysis of AMPK and TORC1 activity in turn, using each as a potential explanatory variable for the other, along with CGG repeat number and ATP steady state levels (because ATP inhibits AMPK), as well as genetic group, gender, and age of the participants. In the regression with AMPK as the dependent variable, the AMPK activity increased at lower TORC1 activities, and the slope of this regression relationship was the same for PM and control cells. This shows that the mutually inhibitory interactions between TORC1 and AMPK are undisturbed in the PM cells. However, because the TORC1 activity is lower in PM cells, this relationship explains some of the elevation of AMPK activity in these cells. In addition to this TORC1-dependent effect on AMPK activity, a further elevation of AMPK was observed in the PM cells that was independent of all the explanatory variables we tested.

Given that the reduced TORC1 activity in PM cells can partly explain the elevated AMPK activity, what might be inhibiting TORC1? With TORC1 as the dependent variable, the CGG repeat number emerged as the most important explanatory variable. Compared to the control cells, the PM cells exhibited a clear decrease in TORC1 activity that was independent of all the other explanatory variables tested (i.e., lower intercept). This was accompanied by a separate repeat number-dependent increase in TORC1 activity that was present both in the control and the PM group (Figure 6b). It is noteworthy that TORC1 signaling is elevated in fragile X syndrome patients, who have CGG repeats larger than 200 bp in length, referred to as the “full mutation” [28,29]. It is possible that the elevated TORC1 activity in fragile X syndrome cells carrying the full mutation is already present as a repeat number-dependent increase in premutation cells, but outweighed by the simultaneous reduction caused by the premutation allele (effect of genetic group in our analysis). This would be consistent with our observation of two concurrent effects in premutation lymphoblasts—a reduction in TORC1 activity that is accompanied by a CGG repeat-dependent elevation. In premutation carriers the reduction dominates, but in cells with the full mutation the elevation dominates the outcome.

Using the observed relationships between the activities of AMPK and TORC1 and between TORC1 activity and the repeat numbers, we were able to predict successfully the effect of CGG repeat numbers on AMPK activity (Figure 6c). This confirmed that the interrelationships between repeat numbers, AMPK, and TORC1 activity in the PM cells do provide a good description of the results with one exception. The elevation of AMPK activity in the PM cells was *not* accompanied by the predicted repeat number-dependent decrease *within* either genetic group. In fact, if anything, the elevated AMPK activity in the PM cells (higher intercept in the premutation genetic group) was accompanied by a further repeat number-dependent increase (Supplementary lasso regression 7). AMPK is known to be activated by diverse cellular stresses [23] that could be proximal causes of AMPK activation in PM cells. These include oxidative stress (but ROS levels were lower in the PM lymphoblasts) and ATP depletion (but steady state ATP levels were higher in the PM cells). Neither of these emerged from the regression analyses as being likely proximal regulators of AMPK in these cells. One possibility is that within the PM group, the AMPK inhibition caused by increases in ATP and decreases in ROS levels is insufficient to overcome activation driven by the premutation alleles (Figure 10). Further work is needed to clarify these issues and the regulatory changes that accompany increasing CGG expansion sizes through the premutation range and into the full mutation range.

Although we are the first to observe reduced TORC1 activity in human cells carrying premutation alleles, these premutation alleles have been modelled in the mouse where TORC1 signaling was found to be reduced [30,31]. As in human cells, this inhibitory effect of the premutation on TORC1 signaling contrasts with the activation observed in an FMR1 knockout mouse, which models the loss of expression of FMR1 in Fragile X cells [28]. While the mechanism by which premutation alleles inhibit TORC1 activity is unknown, there are several possibilities including gain of function RNA toxicity (associated with RNA-mediated protein sequestration by elevated FMR1 mRNA levels) [32,33] and polypeptide toxicity (from accumulation of abnormal, toxic polyG- or polyA-containing RAN translation products) [34]. Exactly how these might in turn inhibit TORC1 signaling is not known, but the cytopathological outcomes of reduced TORC1 signaling could be diverse, including increased autophagy and decreased translation of multiple diverse proteins in the cell [15,16].

Not only did our results indicate a dramatic reduction in TORC1 activity in premutation cells, but they also suggested an additional increase that was correlated with expansion size within the PM group. This could result from the decline in levels of reactive O_2_ species with increasing expansion size in the PM cells (Figure 8). ROS are a cause of oxidative stress which activates AMPK and thereby would inhibit TORC1, while reductive stress is a known activator of TORC1 [35,36]. Thus, the positive correlation between TORC1 activity and repeat number within the premutation group could be caused by decreases in oxidative stress or increases in reductive stress. However, in our multiple regression analyses, the premutation alleles and the expansion size provided the “best” statistical explanation of TORC1 activity. Once these were accounted for, there was no significant additional effect of ROS levels on TORC1 activity. This may be because the reduced oxidative stress we observed does not necessarily mean elevated reductive stress, which we did not measure but in cells is associated with increased ratios of NAD(P)^+^/NAD(P)H. Such increases could occur, for example, if the elevated nonmitochondrial metabolic rate we observed in PM cells provisions the mitochondria with excess NADH. It would be valuable if future studies were to assess directly these ratios and the redox state of PM and control lymphoblasts.

The major source of ROS in cells is leakage of unpaired electrons directly to molecular O_2_, producing superoxide (O_2_^−^) at the point in the electron transport chain where electrons are normally passed from complex I via ubiquinone to complex III. ROS production can be increased by partial blockade of this normal electron flow, so that electrons are forced to divert directly to O_2_. This partial blockade can result from defective downstream electron transport or from a high mitochondrial membrane potential (Δψ_m_) which opposes proton pumping at complexes I, III, and IV. However, our results provided no evidence for any impairment of mitochondrial electron transport or significantly higher Δψ_m_ in PM cells. Not only were the mitochondria in the PM lymphoblasts in this study functionally unimpaired, they were more active in oxidative phosphorylation, confirming the findings in our earlier pilot study [14].

Other studies using peripheral blood mononuclear cells (PBMCs) reported conflicting results. Whereas Alvarez-Mora et al. [12] reported no difference in mitochondrial function between premutation and control PBMCs, Napoli et al. [13] found a generalized reduction in mitochondrial OXPHOS capacity in PBMCs from PM carriers. A recent study by Wang et al. using digitonin-permeabilized PBMCs detected significant reductions only in complex II activity in premutation cells, which were paralleled by alterations in measures of mitochondrial mass and correlated in carriers with changes in white matter hyperintensity and whole brain volumes [37]. Despite their accessibility, ex vivo PBMCs are a mixed population of cell types that are dying and metabolically quiescent with very low mitochondrial respiration rates [38,39], so that differences between premutation and control cells can arise from differences in the cell type mix, can be difficult to measure and may be misleading or unrepresentative of metabolically active cells.

Some of the difficulties of working with PBMCs can be overcome by culturing skin fibroblasts from study participants. In contrast with lymphoblasts, cultured PM fibroblasts have been reported to exhibit lower expression of mitochondrial proteins, accompanied by lower rates of maximum respiration and OXPHOS enzyme activity, as well as elevated ROS levels [11,12,20,37]. Thus, some of the differences between premutation and control cells may depend on the cell type. The physiological state of cultured fibroblasts may represent that of differentiated cells committed to and in different stages of a program of senescence. Lymphoblasts, by contrast, are B cell-derived, metabolically active, proliferating lymphoid cells that may resemble the activated immune cells that drive neuroinflammation in vivo [40,41,42]. Significant resident populations of B cells and their progenitors, that matured during experimentally induced neuroinflammation, were recently found in meninges of the central nervous system of murine models [43]. Resembling activated B cells as they do [42], lymphoblasts may prove to be valuable in vitro models of this newly discovered cell population. Cultured lymphoblasts have been increasingly used in this way to provide powerful biomarkers of diverse neurological diseases (including FXTAS [5,14,18]) as well as cellular models of the underlying disease mechanisms [44,45,46]. Individually or in combination, the elevation in lymphoblasts of mitochondrial respiration, ATP levels, and AMPK activity, as well as the reduction in ROS levels and TORC1 activity could potentially provide biomarkers of FXTAS or its progression. The underlying molecular processes are universally conserved in human cell types and found in all eukaryotes. However, it is emerging that there are clear differences in the pattern of their dysregulation in different neurodegenerative and neurological disorders [14,18,38,39,40]. This needs to be explored more comprehensively in future, larger studies.

## 4. Materials and Methods

### 4.1. Sample Description

Lymphoblasts were derived from 28 male and 5 female controls, as well as 32 male and 11 female PM participants. All but one male PM participant were adults (>31 years of age) at the time of sampling and clinical examination. Samples were collected and clinical examinations performed on two widely separated occasions for 4 PM and 2 control male participants, bringing the total number of separately isolated PM cell lines to 49. At the time of the 2nd sampling, one repeat PM participant had progressed from being clinically unaffected to exhibiting a mild tremor, not diagnosable as FXTAS. Except for one Asian (Chinese) male and one (Thai) female, all participants were white Caucasians. The source of all male and 4 of the female PM participants was a major research project continuing from 2012 at La Trobe University and supported by the National Institutes of Health, USA. This project’s male and female participants were originally recruited through fragile X families’ admissions to the Victorian Genetic Counselling Clinic of the Murdoch Children’s Research Institute, or referred from several neurology clinics associated with the University of Melbourne and Monash University; the minority (some residing in the other Australian states) were self-referred by postings in the community through The Australian Fragile X Association. Sixteen PM carrier males from this cohort were already included in our earlier publication, where basic cellular metabolism parameters were correlated with white matter lesion burden [14], and a further 6 males were included in a study of the relationship between AMPK and clinical and genotypic measures [5]. Thus, of the 32 PM males, 10 were previously unreported. The other source of the female cohort (11 individuals) was an earlier 2008–2010 project supported by a research grant from the National Health and Medical Research of Australia (NHMRC) to ES and DZL. These females, who had originally been ascertained either through their fragile X syndrome (FXS) children diagnosed at the genetic counselling clinics in the states of Victoria and South Australia, or were identified through cascade testing, were incorporated in an earlier study of progression of motor dysfunction based on a larger sample of female PM carriers [18]. All PM participants were classified as belonging to the “FXTAS spectrum” (“FXTAS”), asymptomatic (“Unaffected”) and “Other” categories, as previously described [5]. The latter category comprised individuals presenting with isolated features occurring in FXTAS, such as fibromyalgia, dementia, isolated kinetic tremor, anxiety/depression, and autism. The healthy control group included 23 participants (including 5 females) recruited with funding support (to PRF, DZL, ES, SJA) from the Michael J Fox Foundation as part of a parallel study on Parkinson’s disease (2015-2017). All participants signed informed consent for the present study according to protocols approved by the La Trobe University Human Research Ethics Committee (HEC01-85 and HEC15-058).

### 4.2. Cell culture

#### 4.2.1. PBMC Isolation from Blood and Immortalization

Lymphoblastoid cell lines were created by EBV-mediated transformation of cells from the Peripheral Blood Mononuclear Cell (PBMC) layer at the interface of Ficoll-Paque Plus (Sigma-Aldrich, St. Louis, MO, USA) gradients as previously described [40].

#### 4.2.2. Lymphoblast Culture

Lymphoblasts were cultured in T25 flasks in growth medium (Minimum Essential Medium α (Gibco Life Technologies, Logan, UT, USA), supplemented with 10% FBS and 1% Penicillin/Streptomycin) and were cultured in a humidified 5% CO_2_ incubator at 37 °C. Cells were seeded to at least 2 × 10^5^ cells/mL and fed every three days either by replacing one third of culture medium with fresh medium or split in a 1:3 ratio of cell culture to fresh medium. All experiments were conducted within 15 passages of recovery from frozen storage. Confluent cultures were harvested and resuspended in 250 µL aliquots in Recovery™ Cell Culture Freezing Medium (Gibco Life Technologies, Logan, UT, USA) and stored at −80 °C. Frozen cells were recovered by thawing at 37 C° and seeded into growth medium. Lymphoblast cultures were harvested for experimentation by centrifugation at 500× *g* for 5 min.

### 4.3. Functional Assays

#### 4.3.1. FMR1 CGG Repeat Number

Genomic DNA was isolated from peripheral blood lymphocytes (or immortalized lymphoblasts) using standard methods (Purygene Kit; Gentra, Inc., Minneapolis, MN, USA). For Southern blot analysis, 10 micrograms of isolated DNA were digested with *Eco*RI and *Nru*I. Hybridization was performed using the specific *FMR1* genomic DIG-labelled StB12.3 probe as previously described [47]. 

#### 4.3.2. Mitochondrial Mass and Membrane Potential

Two mitochondrial dyes, MitoTracker Green and MitoTracker Red CMXRos (Thermo Fisher Scientific, Waltham, MA, USA), were used to estimate mitochondrial mass and membrane potential, as originally described by Pendergrass et al. [48] and used previously by us with lymphoblasts [40]. Measurements were made in duplicate for each cell line, averaged, and normalized within every experiment to the values for our standard selected control cell line (C105).

#### 4.3.3. Seahorse Respirometry

Seahorse respirometry was conducted using the Seahorse XFe24 Extracellular Flux Analyzer and Seahorse XF24 FluxPaks (Agilent Technologies, Santa Clara, CA, USA). Oxygen consumption rates (OCR) were measured in lymphoblasts, which had been cultured in 6 well culture plates (corning) in growth medium prior to experiments. Experiments were conducted as described previously [40] using 8 × 10^5^ cells/well for each lymphoblast cell line. We measured the basal O_2_ consumption rate (basal OCR), the decrease in OCR after oligomycin addition (OCR attributable to ATP synthesis), the maximum OCR after uncoupling electron transport with the protonophore CCCP (carbonyl cyanide *m*-chlorophenyl hydrazone), the subsequent decrease after blockade with rotenone (Complex I OCR) and antimycin A (“nonmitochondrial” OCR), and the “proton leak” (difference between OCR after oligomycin treatment and the “nonmitochondrial” OCR) in pmol/min/well. The results were averaged over 4 replicate wells per experiment and at least 3 independent experiments per cell line.

#### 4.3.4. AMPK Activity

AMPK assays were performed as described by us previously [49]. Lysates were prepared from confluent cell lines (~25 mL) grown in T75 flasks, harvested, lysed in lysis buffer supplemented with phosphatase inhibitors (50 mM Tris-HCl pH 7.4, 150 mM NaCl, 1 mM EDTA, 1 mM EGTA, 1% Triton X-100, 50 mM NaF, 5 mM sodium pyrophosphate), and then snap-frozen in liquid nitrogen. Thawed lysates were cleared by centrifugation at 10,000× *g* for 5 min. Supernatant total protein concentrations were determined with the Pierce^TM^ BCA Protein Assay Kit (Thermo Fisher Scientific, Waltham, MA, USA). To concentrate the AMPK protein, one mg of total supernatant protein was immunoprecipitated with rabbit polyclonal anti-AMPKα1 antibody α1-(339–358) [49] bound to equilibrated protein A-agarose beads. The beads were recovered and washed four times by centrifugation before being resuspended in 60 μL wash buffer (50 mM HEPES pH 7.4, 150 mM NaCl, 10% glycerol, 0.1% Tween-20). This was named the AMPK slurry. AMPK activity was assayed over 10 min at 30 °C by adding 20 μL of the AMPK slurry to 15 μL buffer (5 mM MgCl_2_, 50 mM HEPES pH 7.4, 0.1% Tween-20, and 1 mM DTT) containing 100 μM SAMS synthetic peptide (NH2-HMRSAMSGLHLVKRR-COOH). Reactions were started by adding [γ-^32^P]-ATP (final concentration 200 μM) and stopped by spotting 21 μL onto P81 ion-exchange chromatography paper (Whatman, GE Healthcare). Liquid scintillation counting (Perkin Elmer, Waltham, MA, USA) was used to measure the incorporation of ^32^P into the SAMS peptide. Duplicates were averaged and normalized against the average value from all the control cell lines, in each independent experiment.

#### 4.3.5. TORC1 Activity

TORC1 activity in ME/CFS lymphoblast lysates was measured using a time-resolved FRET-based multiwell plate assay based on the phosphorylation state of 4E-BP1, a major TORC1 substrate (Cisbio Bioassays, Codolet, France). Lymphoblasts were plated in duplicate wells for each cell line in growth medium at 5 × 10^4^ cells/well in a 384-well plate. Lysis buffer was added to each well as per the manufacturer’s instructions and the plate mixed on an orbital shaker for 40 min at RT. Lysates from each sample were then transferred to a white-bottom, white-sided 384 well plate (Corning, New York, NY, USA), including various controls according to the manufacturer’s instructions. Freshly prepared antibody mix was then added to each well (anti-4E-BP1 antibody labelled with d2 acceptor, and anti-phospho-4E-BP1 antibody labelled with Eu^3+^-cryptate donor). The plate was incubated at RT for 2 h and scanned using a Clariostar plate reader (BMG, Ortenberg, Germany) by reading the fluorescence emission at two different wavelengths (665 nm and 620 nm). The ratio of the FRET signal from anti-phospho-4E-BP1 antibody to the donor fluorescence signal from anti-4E-BP1 antibody was measured.

#### 4.3.6. Reactive Oxygen Species (ROS)

Intracellular ROS levels were measured using the Fluorometric Intracellular ROS Kit (MAK145-1KT, Sigma, St. Louis, MO, USA). Lymphoblasts were seeded in triplicate in 90 µL of Dulbecco’s phosphate-buffered saline (Sigma, St. Louis, MO, USA) at 1.25 × 10^5^ cells/well into a 96 well black, clear flat bottom plate. Fresh reaction mixture was prepared according to the manufacturer’s instructions; 100 µL was added to duplicate wells for each cell line, and 100 uL of PBS was added to the remaining well to use for background subtraction. A cell-free control well containing PBS and reaction mix was also included. The plate was incubated in darkness for 1 h at 37 °C with 5% CO_2_. The fluorescence was then read on a Clariostar microplate reader (excitation = 520, emission = 605 nm). A control cell line (C105) was included in each experiment to allow internal normalization to control for between experiment variation.

#### 4.3.7. ATP Steady State Levels

Steady state ATP levels were measured using luciferase ATP-driven luminescence as per the manufacturer’s instructions using the ATP Determination Kit (Invitrogen Molecular Probes, ThermoFisher Scientific, Waltham, MA, USA, 02451), as described previously [40]. The signal was normalized against that from a control cell line C105 used in every experiment as an internal control.

### 4.4. Statistical Analysis

All statistical analysis was conducted using the WinStat (R. Fitch Software, Cambridge, MA 02141, USA, https://www.winstat.com, Version 2012.1.0.96) addon for Excel and statistics packages in R [50], R Commander [51], and StatsNotebook [52].

#### 4.4.1. Two- and Multi-Sample Tests

Two-sample tests were performed using Student’s *t*-tests and Mann–Whitney U tests, while multisample tests used ANOVA coupled with least squares difference tests for pairwise comparisons within the ANOVA. The χ^2^ test was used to test for differences in the distribution of sample measurements (CGG repeat numbers, respiration rates) between different PM phenotypic groups (“Unaffected”, FXTAS, “Other”).

#### 4.4.2. Correlation and Linear Regression Analysis

Correlations were calculated using the Pearson parametric and the Spearman ranked correlation coefficients. Multiple regressions were conducted using generalized log–linear or log–log models, incorporating additional dummy variables that allowed the intercepts and slopes of regression lines for subgroups (genetic group and participant gender combinations) to differ. Outliers were detected as observations lying outside the 95% data confidence limits for simple linear regressions between the variables. Final regression models were arrived at after automated stepwise removal of insignificant variables with the significance cut-off for variable removal set at 0.05. Details of each multiple regression reported are given in the Supplemental Information. Additionally, lasso regressions (using the elastic net regression package, glmnet [53], in R) and robust regressions (using StatsNotebook [52] and the robustbase package in R [50]) and were used to verify the results using techniques less sensitive than traditional methods to the presence of outliers and leverage points.

## Figures and Tables

**Table 1:** Spearman rank correlations amongst key parameters of mitochondrial activity in control cells. Spearman rank correlations within the control and the PM genetic groups are shown. Significant correlations (*p* < 0.05) are highlighted in red, while insignificant correlations (*p* > 0.1) are highlighted in green. OCR = O_2_ consumption rate in pmol/min. Similar results were obtained using the Pearson correlation coefficient (Supplementary Table S1). The table shows one-sided significances for correlations in the directions indicated by the sign of the correlation coefficient. In this case, all correlations are expected to be positive, so in those cases where an insignificant negative correlation was observed, the significance in a one-sided test for a positive correlation is *p* = 1 − a, where a is the value shown in the table.

Spearman Rank Correlations Amongst Mitochondrial Respiratory Activities and ATP Levels		Premutation Lymphoblasts						
		Basal OCR	OCR by ATP Synthesis	Maximum OCR	Complex I OCR	“Nonmitochondrial” OCR	“Proton Leak” OCR	ATP Steady State Levels
**Control lymphoblasts**	**Basal OCR**							
	Correlation coefficient		0.896	0.700	0.699	0.771	0.505	0.328
	Valid cases		44	44	44	44	42	43
	One-sided significance		1.09 × 10^−16^	6.31 × 10^−8^	6.47 × 10^−8^	4.61 × 10^−10^	3.23 × 10^−4^	1.58 × 10^−2^
	**OCR by ATP synthesis**							
	Correlation coefficient	0.925		0.641	0.656	0.524	0.336	0.338
	Valid cases	33		44	44	44	42	43
	One-sided significance	7.46 × 10^−15^		1.37 × 10^−6^	6.75 × 10^−7^	1.30 × 10^−4^	1.47 × 10^−2^	1.33 × 10^−2^
	**Maximum OCR**							
	Correlation coefficient	0.890	0.785		0.989	0.592	0.401	0.279
	Valid cases	33	33		44	44	42	43
	One-sided significance	2.11 × 10^−12^	3.09 × 10^−8^		1.86 × 10^−36^	1.14 × 10^−5^	4.27 × 10^−3^	3.51 × 10^−2^
	**Complex I OCR**							
	Correlation coefficient	0.876	0.789	0.995		0.570	0.411	0.297
	Valid cases	33	33	33		44	42	43
	One-sided significance	1.23 × 10^−11^	2.38 × 10^−8^	1.94 × 10^−32^		2.65 × 10^−5^	3.41 × 10^−3^	2.63 × 10^−2^
	**“Nonmitochondrial” OCR**							
	Correlation coefficient	0.817	0.633	0.812	0.777		0.418	0.188
	Valid cases	33	33	33	33		42	43
	One-sided significance	3.45 × 10^−9^	3.90 × 10^−5^	4.80 × 10^−9^	5.14 × 10^−8^		2.92 × 10^−3^	1.14 × 10^−1^
	**“Proton leak” OCR**							
	Correlation coefficient	0.688	0.521	0.700	0.697	0.651		−0.014
	Valid cases	31	31	31	31	31		31
	One-sided significance	9.35 × 10^−6^	1.33 × 10^−3^	5.75 × 10^−6^	6.56 × 10^−6^	3.68 × 10^−5^		4.70 × 10^−1^
	**ATP steady state levels**							
	Correlation coefficient	−0.055	−0.047	0.054	0.082	0.059	0.097	
	Valid cases	33	33	33	33	33	41	
	One-sided significance	3.80 × 10^−1^	3.98 × 10^−1^	3.83 × 10^−1^	3.26 × 10^−1^	3.72 × 10^−1^	2.74 × 10^−1^	

## Data Availability

Ethics approvals and informed consent documents permit the data obtained in this work to be published only in deidentified, aggregated form. All aggregated data is included in this manuscript and the Supplementary Information.

## Supplementary Materials

The following are available online at https://www.mdpi.com/article/10.3390/ijms221910393/s1.
