# Supplementary material for: Relationships between Mitochondrial Function, AMPK, and TORC1 Signaling in Lymphoblasts with Premutation Alleles of the FMR1 Gene
Source: Int J Mol Sci. 2021 Sep 27;22(19):10393. doi: 10.3390/ijms221910393 (PMC8508849; doi:10.3390/ijms221910393)
Supplement: Supplementary file 1 [file ijms-22-10393-s001.zip › ijms-1382790-supplementary.pdf]

Supplementary information

Contents

Supplementary tables and figures 2

Supplementary Figure S1. Mitochondrial measures that are not significantly altered in lymphoblasts carrying PM alleles of FMR1. 2

Supplementary Table S1. Pearson correlations amongst key parameters of mitochondrial activity. 3

Multiple regressions 4

Supplementary regression 1. Basal respiration rates depend on AMPK activity and gender. 4

Supplementary regression 2. O_2 consumption by ATP synthesis at Complex V depends on AMPK activity and gender. 5

Supplementary regression 3. The "proton leak" in PM lymphoblasts is elevated, but not significantly related to the gender, age, CGG expansion size, AMPK or TORC1 activity in the participants. 6

Supplementary regression 4. Elevated "nonmitochondrial" O_2 consumption in PM lymphoblasts can be explained by decreased TORC1 activity. 7

Supplementary regression 5. AMPK activity in PM lymphoblasts can be explained by decreased TORC1 activity as well as a TORC1-independent component. 8

Supplementary regression 6. TORC1 activity is reduced in PM lymphoblasts, but is also positively correlated with expansion size within both the control and PM groups. 9

Supplementary regression 7. The elevated ATP steady state levels in PM cells depend on AMPK activity. 10

Supplementary regression 8. Reactive O_2 species levels are reduced in PM cells, dependent on the CGG expansion size. 11

Lasso regressions. 12

Supplementary lasso regression 1. Basal OCR dependence on AMPK,TORC1, CGG, genetic group, age and gender. 12

Supplementary lasso regression 2. ATP synthesis OCR dependence on AMPK,TORC1, CGG, genetic group, age and gender. 13

Supplementary lasso regression 3. Proton leak dependence on AMPK,TORC1, CGG, genetic group, age and gender 13

Supplementary lasso regression 4. Nonmitochondrial OCR dependence on AMPK, CGG, TORC1, genetic group, age and gender. 14

Supplementary lasso regression 5. AMPK activity dependence on TORC1, CGG expansion size, ATP steady state levels, genetic group, age and gender. 14

Supplementary lasso regression 6. TORC1 activity dependence on AMPK, CGG, ATP levels, genetic group, age and gender 15

Supplementary lasso regression 7. Lasso multivariate regression of AMPK and TORC1 activities against CGG expansion size. 16

Supplementary lasso regression 8. ATP steady state level dependence on AMPK, TORC1, CGG, ATP synthesis OCR, nonmitochondrial OCR, basal OCR, genetic group, age and gender. 17

Supplementary lasso regression 9. ROS dependence on AMPK, TORC1, CGG, basal OCR, genetic group, age and gender. 17

Supplementary tables and figures

Supplementary Figure S1. Mitochondrial measures that are not significantly altered in lymphoblasts carrying PM alleles of FMR1.

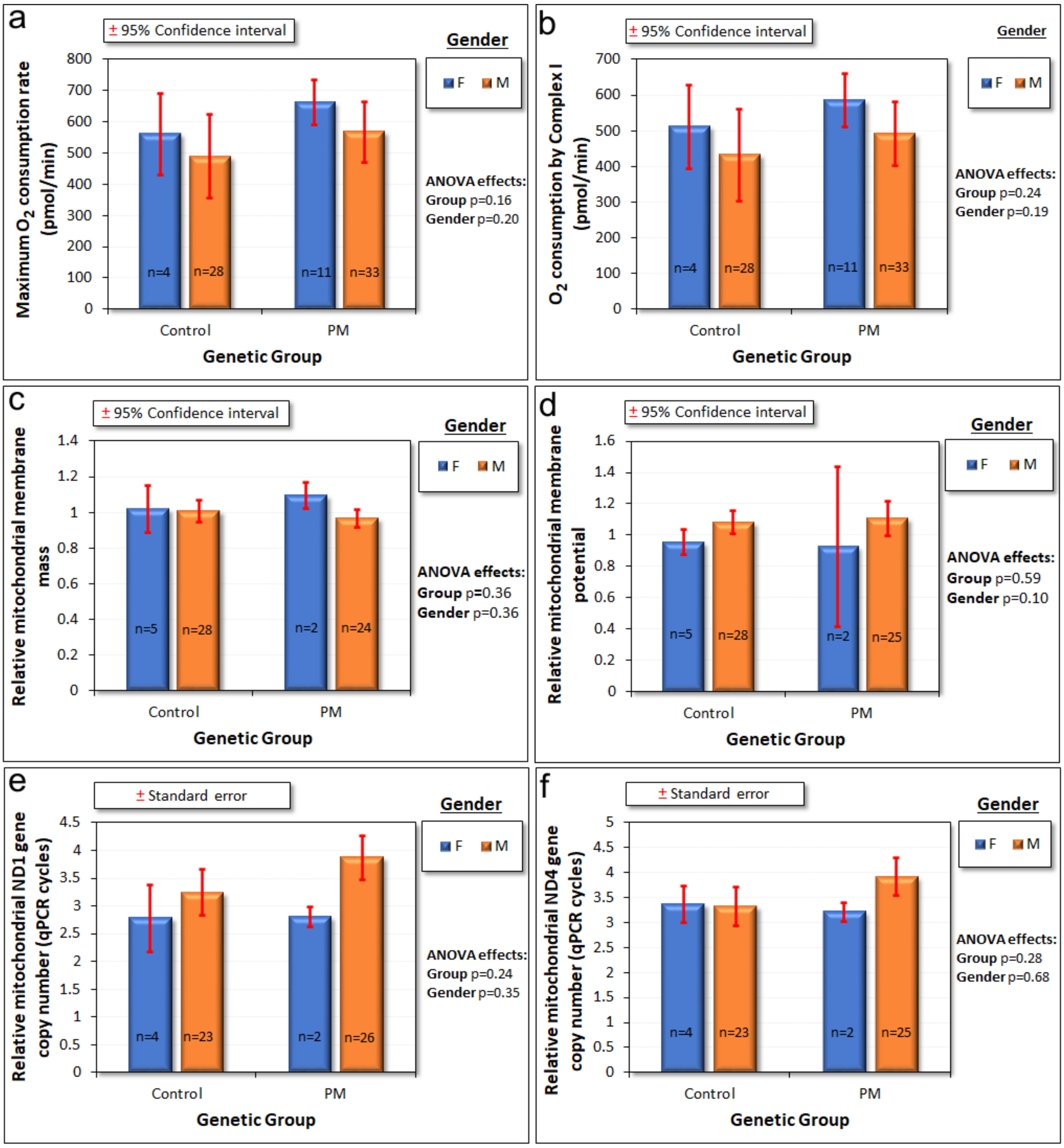

Supplementary Figure S1. Mitochondrial measures that are not significantly altered in lymphoblasts carrying PM alleles of FMR1. Statistical significances shown were derived from 2-way ANOVA using the average of multiple, independent assays for each of the indicated number of lymphoblast cell lines. Identical conclusions concerning the effect of the PM allele were obtained using 2-sample t and Mann-Whitney U tests on pooled data form males and females. The maximum uncoupled electron transport rate (a) and its major contributor, complex I (b) showed the same trend as other parameters of mitochondrial activity (Results Figure 3) but this did not reach statistical significance. To facilitate comparisons across all measures of mitochondrial function, the results for both males and females are shown, but particularly for panels c-f the female sample sizes were too small to allow detection of anything but large effects of gender. The analysis of variance showed no significant effect of either genetic group and gender on any of these measures.

Supplementary Table S1. Pearson correlations amongst key parameters of mitochondrial activity.

Pearson correlations amongst mitochondrial respiratory activities and ATP levels		Premutation lymphoblasts						
		Basal OCR	OCR by ATP synthesis	Maximum OCR	Complex I OCR	"Nonmitochondrial" OCR	"Proton leak" OCR	ATP steady state levels
Control lymphoblasts	**Basal OCR**							
	Correlation coefficient		0.933	0.741	0.757	0.678	0.549	0.394
	Valid cases		44	44	44	44	44	43
	One-sided significance		1.56E-20	4.39E-09	1.42E-09	2.12E-07	5.71E-05	4.47E-03
	**OCR by ATP synthesis**							
	Correlation coefficient	0.967		0.674	0.699	0.469	0.326	0.301
	Valid cases	33		44	44	44	44	43
	One-sided significance	3.32E-20		2.63E-07	6.60E-08	6.57E-04	1.53E-02	2.49E-02
	**Maximum OCR**							
	Correlation coefficient	0.872	0.789		0.986	0.655	0.282	0.272
	Valid cases	33	33		44	44	44	43
	One-sided significance	2.05E-11	2.47E-08		1.62E-34	6.99E-07	3.19E-02	3.90E-02
	**Complex I OCR**							
	Correlation coefficient	0.862	0.788	0.998		0.618	0.273	0.312
	Valid cases	33	33	33		44	44	43
	One-sided significance	5.70E-11	2.57E-08	4.56E-38		3.83E-06	3.64E-02	2.10E-02
	**"Nonmitochondrial" OCR**							
	Correlation coefficient	0.762	0.661	0.767	0.731		0.430	0.250
	Valid cases	33	33	33	33		44	43
	One-sided significance	1.29E-07	1.39E-05	9.88E-08	6.91E-07		1.78E-03	5.29E-02
	**"Proton leak" OCR**							
	Correlation coefficient	0.754	0.630	0.712	0.698	0.549		0.372
	Valid cases	32	32	32	32	32		43
	One-sided significance	3.09E-07	5.50E-05	2.42E-06	4.39E-06	5.63E-04		7.00E-03
	**ATP steady state levels**							
	Correlation coefficient	-0.073	-0.093	0.103	0.121	-0.022	-0.070	
	Valid cases	33	33	33	33	33	32	
	One-sided significance	3.44E-01	3.03E-01	2.84E-01	2.52E-01	4.53E-01	3.52E-01	

Supplementary Table S1. Pearson correlations amongst key parameters of mitochondrial activity.

Pearson correlations amongst components of the mitochondrial respiratory activities and ATP steady state levels in control and PM lymphoblasts are shown. Significant correlations ($p<0.05$) are highlighted in red, correlations suggesting a trend that did not reach statistical significance ($0.05<p<0.1$) are highlighted in amber, while insignificant correlations ($p>0.1$) are highlighted in green. OCR=O_2 consumption rate in pmol/min. Similar results were obtained using the Spearman rank correlation coefficient.

Multiple regressions

Multiple regressions involved the indicated variables with stepwise, backward removal of variables with insignificant regression coefficients ($p<0.05$). Dummy variables were included to allow intercepts to differ from the controls in the PM group (“Genetic Group code”), the female cohort compared to the male cohort (“Gender code”) and the female cohort in the PM group compared to the other groups (“Gender.PM”). Variables whose names contain “PM”, “PM.slope” or “PM.Slope” are dummy variables providing additional slope terms for the indicated variables, thereby allowing the slopes for the those variables to differ in the PM group from controls.

Supplementary regression 1. Basal respiration rates depend on AMPK activity and gender.

Multiple Regression

X-Variables: Genetic Group Code
Log(CGG)
Log(CGG).PM.slope
Patient.age
Patient.age PM.Dummy.slope
Log(AMPK)
log AMPK.PM.Slope
Log(TORC1 activity) (minus outliers and 1.2.19 expt)
LogTORC1.PM.slope
Gender code
Gender.PM

Y-Variable: Basal.average..pmol.min.

Method: Backward

Steps	P	R-Square	Corrected
Gender.PM (-)	0.968915399	0.26173919	0.062209241
Patient.age (-)	0.949756441	0.261658882	0.086788618
LogTORC1.PM.slope (-)	0.948719862	0.261577444	0.110106151
log AMPK.PM.Slope (-)	0.803166677	0.26038502	0.130952398
Log(CGG) (-)	0.577793204	0.254561601	0.145473055
Log(CGG).PM.slope (-)	0.425492816	0.242781089	0.15263598
Log(TORC1 activity) (minus ou	0.506969361	0.234704858	0.163514612
Genetic Group Code (-)	0.270645012	0.212541128	0.15885075
Patient.age PM.Dummy.slope	0.465010686	0.202819111	0.16738885

Summary

	N	R	R-Square	Std.Error
**normal**	48	0.450354429	0.202819111	127.4400952
**corrected**		0.409131824	0.16738885	

Equation

95%

	Coefficient	Conf. (±)	Std.Error	T	P
Constant	362.7274685	43.28469317	21.4908001	16.8782673	4.34224E-21
Log(AMPK)	243.0356072	205.2491552	101.9059682	2.384900625	0.021357354
Gender code	100.8179791	88.22008945	43.80117237	2.301718737	0.026034533

Analysis of variance

	Sum of Squares	Degrees of Freedom	Mean Square	F	P
**Regression**	185941.6519	2	92970.82596	5.724459868	0.006095787
**Residue**	730844.0035	45	16240.97786		
**Total**	916785.6555	47	19506.07778		

Supplementary regression 2. O_2 consumption by ATP synthesis at Complex V depends on AMPK activity and gender.

Multiple Regression

X-Variables: Genetic Group Code
Log(CGG)
Log(CGG).PM.slope
Patient.age
Patient.age PM.Dummy.slope
Log(AMPK)
log AMPK.PM.Slope
Log(TORC1 activity) (minus outliers and 1.2.19 expt)
LogTORC1.PM.slope
Gender code
Gender.PM

Y-Variable: ATP.average..pmol.min.

Method: Backward

Steps	P	R-Square	Corrected
log AMPK.PM.Slope (-)	0.913609071	0.301101776	0.112210363
Log(TORC1 activity) (minus ou	0.9055022	0.300831899	0.135239454
Log(CGG) (-)	0.884092135	0.300435573	0.156935177
Gender.PM (-)	0.797721205	0.299241066	0.176608253
Patient.age PM.Dummy.slope	0.706020626	0.2967125	0.193792378
Log(CGG).PM.slope (-)	0.619474384	0.29241743	0.20818141
Genetic Group Code (-)	0.803012313	0.291355755	0.225435361
LogTORC1.PM.slope (-)	0.730643164	0.289376908	0.240925333
Patient.age (-)	0.153564282	0.255321408	0.222224582

Summary

	N	R	R-Square	Std.Error
**normal**	48	0.505293388	0.255321408	89.75395554
**corrected**		0.471407023	0.222224582	

Equation

95%

	Coefficient	Conf. (±)	Std.Error	T	P
Constant	234.5419972	30.48469496	15.13561579	15.4960327	1.16794E-19
Log(AMPK)	165.2566373	144.5535922	71.77069135	2.302564379	0.025982705
Gender code	95.21232416	62.13195287	30.84844273	3.0864548	0.003461083

Analysis of variance

	Sum of Squares	Degrees of Freedom	Mean Square	F	P
**Regression**	124290.5388	2	62145.2694	7.714377376	0.001316132
**Residue**	362509.7641	45	8055.772536		
**Total**	486800.3029	47	10357.45325		

Supplementary regression 3. The "proton leak" in PM lymphoblasts is elevated, but not significantly related to the gender, age, CGG expansion size, AMPK or TORC1 activity in the participants.

Multiple Regression

X-Variables: Genetic Group Code
Log(CGG)
Log(CGG).PM.slope
Patient.age
Patient.age PM.Dummy.slope
Log(AMPK)
log AMPK.PM.Slope
Log(TORC1 activity) (minus outliers and 1.2.19 expt)
LogTORC1.PM.slope
Gender code
Gender.PM

Y-Variable: Proton.leak.average..pmol.min.

Method: Backward

Steps	P	R-Square	Corrected
Log(TORC1 activity) (minus ou	0.998185921	0.191302996	0
Patient.age (-)	0.86423079	0.190719012	0.017301657
log AMPK.PM.Slope (-)	0.838617644	0.189909811	0.039195357
Gender.PM (-)	0.789528515	0.188550717	0.059456513
Patient.age PM.Dummy.slope	0.669179745	0.185138267	0.076490036
Log(CGG).PM.slope (-)	0.619026468	0.180598675	0.091533314
Log(AMPK) (-)	0.48826096	0.171902812	0.101426456
LogTORC1.PM.slope (-)	0.130222929	0.130107636	0.075739363
Log(CGG) (-)	0.149936662	0.091308825	0.054219389
Gender code (-)	0.181105154	0.057176485	0.038320015

Summary

	N	R	R-Square	Std.Error
**normal**	52	0.23911605	0.057176485	51.08094236
**corrected**		0.195754988	0.038320015	

Equation

95%

	Coefficient	Conf. (±)	Std.Error	T	P
Constant	52.62646347	20.5198184	10.21618847	5.151281577	4.39937E-06
Genetic Group Code	24.68807419	28.47693886	14.17779479	1.741319758	0.087777725

Analysis of variance

	Sum of Squares	Degrees of Freedom	Mean Square	F	P
**Regression**	7911.791923	1	7911.791923	3.032194499	0.087777725
**Residue**	130463.1336	50	2609.262672		
**Total**	138374.9255	51	2713.233834		

Supplementary regression 4. Elevated “nonmitochondrial” O_2 consumption in PM lymphoblasts can be explained by decreased TORC1 activity.

Multiple Regression

X-Variables: Genetic Group Code
Log(CGG)
Log(CGG).PM.slope
Patient.age
Patient.age PM.Dummy.slope
Log(AMPK)
log AMPK.PM.Slope
Log(ATP.steady.state)
Log(ATPsteady).PM.slope
Log(TORC1 activity) (minus outliers and 1.2.19 expt)
LogTORC1.PM.slope
Gender code
Gender.PM

Y-Variable: Nonrespiratory.average..pmol.min.

Method: Backward

Steps	P	R-Square	Corrected
Gender code (-)	0.855199062	0.301367541	0.080746764
Patient.age PM.Dummy.slope	0.659240509	0.297735882	0.099661387
Log(ATP.steady.state) (-)	0.502857936	0.28950055	0.111875688
log AMPK.PM.Slope (-)	0.528899994	0.28233323	0.124796622
Log(AMPK) (-)	0.660572561	0.278908402	0.141557621
Log(CGG) (-)	0.376923973	0.265215874	0.145599853
Genetic Group Code (-)	0.669651102	0.262062571	0.16143474
Patient.age (-)	0.337626288	0.246300766	0.162556407
Log(CGG).PM.slope (-)	0.356206991	0.231745991	0.164941295
Log(ATPsteady).PM.slope (-)	0.097382494	0.183924597	0.131834678
LogTORC1.PM.slope (-)	0.065444714	0.122145183	0.085567899
Gender.PM (-)	0.128107248	0.078300334	0.059490137

Summary

	N	R	R-Square	Std.Error
**normal**	51	0.279821969	0.078300334	31.05311176
**corrected**		0.243906	0.059490137	

Equation

95%

	Coefficient	Conf. (±)	Std.Error	T	P
Constant	72.16326618	16.25259456	8.087577122	8.922729897	7.68824E-12
Log(TORC1 activity)	-53.85338075	53.04349182	26.39537518	-2.040258204	0.046732988

Analysis of variance

	Sum of Squares	Degrees of Freedom	Mean Square	F	P
**Regression**	4014.029117	1	4014.029117	4.162653539	0.046732988
**Residue**	47250.49176	49	964.2957501		
**Total**	51264.52087	50	1025.290417		

Supplementary regression 5. AMPK activity in PM lymphoblasts can be explained by decreased TORC1 activity as well as a TORC1-independent component.

Multiple Regression

X-Variables: Genetic Group Code
Log(CGG)
Log(CGG).PM.slope
Patient.age
Patient.age PM.Dummy.slope
Log(ATP.steady.state)
Log(ATPsteady).PM.slope
Log(TORC1 activity) (minus outliers and 1.2.19 expt)
LogTORC1.PM.slope
Gender code

Y-Variable: AMPK (minus outliers)

Method: Backward

Steps	P	R-Square	Corrected
Log(CGG) (-)	0.925374914	0.391105932	0.250591916
Patient.age PM.Dummy.slope	0.890911862	0.390808392	0.26897007
Gender code (-)	0.555627209	0.385428498	0.280501656
Log(CGG).PM.slope (-)	0.50072605	0.378510328	0.28972609
LogTORC1.PM.slope (-)	0.326830149	0.363945822	0.289986034
Patient.age (-)	0.227354075	0.341759387	0.281919331
Log(ATP.steady.state) (-)	0.133789482	0.306852979	0.260643178
Log(ATPsteady).PM.slope (-)	0.33360599	0.292138651	0.261362071

Summary

	N	R	R-Square	Std.Error
**normal**	49	0.540498521	0.292138651	0.440097554
**corrected**		0.511235827	0.261362071	

Equation

	Coefficient	95% Conf. (±)	Std.Error	T	P
Constant	0.809708564	0.234022342	0.116261537	6.964543751	1.02994E-08
Genetic Group Code	0.361824015	0.279178205	0.138694823	2.608778093	0.012216468
Log TORC1 activity	-0.843425162	0.822227208	0.408479799	-2.064790386	0.044606317

Analysis of variance

	Sum of Squares	Degrees of Freedom	Mean Square	F	P
**Regression**	3.677024817	2	1.838512408	9.492238827	0.000353841
**Residue**	8.909549404	46	0.193685857		
**Total**	12.58657422	48	0.262220296		

Supplementary regression 6. TORC1 activity is reduced in PM lymphoblasts, but is also positively correlated with expansion size within both the control and PM groups.

Multiple Regression

X-Variables: Genetic Group Code
Log(CGG)
Log(CGG).PM.slope
Patient.age
Log(AMPK)
log AMPK.PM.Slope
Log(ATP.steady.state)
Log(ATPsteady).PM.slope
Gender code
Gender.PM

Y-Variable: Log(TORC1 activity)

Method: Backward

Steps	P	R-Square	Corrected
Gender code (-)	0.865073782	0.383587775	0.2413388
Log(CGG) (-)	0.768201335	0.382195558	0.25863467
Gender.PM (-)	0.498256663	0.374980886	0.268270306
log AMPK.PM.Slope (-)	0.348502292	0.361270272	0.270023167
Log(ATP.steady.state) (-)	0.260662541	0.341499128	0.264929259
Log(ATPsteady).PM.slope (-)	0.106028654	0.299757281	0.236098852
Log(AMPK) (-)	0.061899037	0.241344394	0.190767354
Patient.age (-)	0.108540435	0.196148892	0.161198844

Summary

	N	R	R-Square	Std.Error
**normal**	49	0.442886997	0.196148892	0.143734084
**corrected**		0.401495758	0.161198844	

Equation

95%

	Coefficient	Conf. (±)	Std.Error	T	P
Constant	-0.194068235	0.057864342	0.028746817	-6.750946944	2.15547E-08
Genetic Group Code	-1.430947476	1.294391866	0.643049663	-2.225251885	0.031007842
Log(CGG).PM.slope	0.698363411	0.68215382	0.338891796	2.060726813	0.045009582

Analysis of variance

	Sum of Squares	Degrees of Freedom	Mean Square	F	P
**Regression**	0.231892983	2	0.115946492	5.612263858	0.006592333
**Residue**	0.950336397	46	0.020659487		
**Total**	1.182229381	48	0.024629779		

Supplementary regression 7. The elevated ATP steady state levels in PM cells depend on AMPK activity.

Multiple Regression

X-Variables: Genetic Group Code
Log(CGG)
Log(CGG).PM.slope
Log(AMPK)
log AMPK.PM.Slope
Log(Basal)
log(Basal).PM.slope
log(ATP.average)
log(ATP.average).dummy.slope
Log(Nonrespiratory.average)
Log(Nonrespiratory.average).PM.slope
Log(TORC1 activity)
LogTORC1.PM.slope
Gender code
Gender.PM

Y-Variable: ATP.average..nM.10.6cells.normalised.to.C101.

Method: Backward

Steps	P	R-Square	Corrected
Log(Basal) (-)	0.969550463	0.462391891	0.241023846
Log(CGG).PM.slope (-)	0.958539094	0.462348522	0.262649402
Genetic Group Code (-)	0.959123451	0.462307587	0.283076782
Log(Nonrespiratory.average) (-)	0.920821658	0.462157934	0.302258941
log(ATP.average) (-)	0.89417475	0.461897148	0.320291135
Gender code (-)	0.590689739	0.457730674	0.332591599
Log(CGG) (-)	0.519269973	0.4518495	0.3422194
Log(Nonrespiratory.average).PM.slope (-)	0.367632883	0.4404707	0.344941308
log(Basal).PM.slope (-)	0.313882732	0.426282581	0.344322949
Log(TORC1 activity) (-)	0.159109738	0.398211338	0.328235912
Log(AMPK) (-)	0.308449736	0.383344104	0.327284477
LogTORC1.PM.slope (-)	0.149138807	0.35312974	0.310005056
Gender.PM (-)	0.051325402	0.295510529	0.264880552
log(ATP.average).dummy.slope (-)	0.083565351	0.247597729	0.23158917

Summary

	N	R	R-Square	Std.Error
**normal**	49	0.49759193	0.247597729	0.43474265
**corrected**		0.481237125	0.23158917	

Equation

95%

	Coefficient	95% Conf. (±)	Std.Error	T	P
Constant	0.959287979	0.140056316	0.069619474	13.77901794	4.00985E-18
log AMPK.PM.Slope	1.842399161	0.942450347	0.468475106	3.932757869	0.000275115

Analysis of variance

	Sum of Squares	Degrees of Freedom	Mean Square	F	P
**Regression**	2.923202584	1	2.923202584	15.46658446	0.000275115
**Residue**	8.883055068	47	0.189001172		
**Total**	11.80625765	48	0.245963701		

Supplementary regression 8. Reactive O_2 species levels are reduced in PM cells, dependent on the CGG expansion size.

Multiple Regression

X-Variables: Log(CGG)
Log(CGG).PM.slope
Patient.age
Patient.age PM.Dummy.slope
Log(AMPK)
log AMPK.PM.Slope
Log(Basal)
log(Basal).PM.slope
Log(TORC1 activity) (minus outliers and 1.2.19 expt)
LogTORC1.PM.slope
Gender code
Gender.PM

Y-Variable: ROS.average..Fluorescence.relative.to.C105.

Method: Backward

Steps	P	R-Square	Corrected
Log(TORC1 activity) (minus	0.976545539	0.438880344	0.114021596
log(Basal).PM.slope (-)	0.973829383	0.438847713	0.158271569
Gender code (-)	0.96897564	0.438804195	0.198291707
log AMPK.PM.Slope (-)	0.944968472	0.438673784	0.23455516
Log(Basal) (-)	0.915797852	0.438381937	0.2674547
Patient.age PM.Dummy.slo	0.840468814	0.437369887	0.296712358
LogTORC1.PM.slope (-)	0.865350679	0.436681228	0.324017473
Patient.age (-)	0.846256981	0.435816321	0.349018831
Log(AMPK) (-)	0.813991416	0.434590472	0.371767191
Log(CGG) (-)	0.528746269	0.426062282	0.385066731
Gender.PM (-)	0.094764424	0.364762925	0.342858198

Summary

	N	R	R-Square	Std.Error
**normal**	31	0.603956062	0.364762925	0.255591679
**corrected**		0.585540945	0.342858198	

Equation

95%

	Coefficient	Conf. (±)	Std.Error	T	P
Constant	1.079679619	0.111374187	0.054455589	19.82679159	2.07802E-18
Log(CGG).PM.slope	-0.219579773	0.110052013	0.053809122	-4.08071651	0.000321186

Analysis of variance

	Sum of Squares	Degrees of Freedom	Mean Square	F	P
**Regression**	1.087843128	1	1.087843128	16.65224724	0.000321186
**Residue**	1.894486087	29	0.065327106		
**Total**	2.982329214	30	0.099410974		

Lasso regressions.

Lasso regression is a machine learning regression method that combines variable (regression coefficient) selection with determination of the best-fitting regression for a set of dependent (Y) variables (typically one) and independent (explanatory X) variables. It numerically finds the regression coefficients that minimize both the regression errors and the number of explanatory variables in a regression model. In each lasso regression (see below), a regression penalty (λ) is applied to the regression coefficients, reducing them and their influence on the outcome. As a result, lasso regression tends to underestimate individual regression coefficients, but it is less sensitive than ordinary least squares regression to the effect of individual outliers. The regression penalties were applied over an automatically determined range of values for λ to find the value at which the regression error is minimized. The plot of Mean Squared Error versus Log(λ) shows on the X axis the logarithm of 2000 values of the lasso regression penalty applied to regression coefficients and on the Y axis the mean squared error for the regression using those coefficients. At each successive value of λ, the data was subdivided randomly into 5 equal subsets ("folds") and each subset was, in turn, put aside as a test set for cross validation, while the other 4 fractions (80% of the data) were used as the training set. For each of the 5 test sets in turn, the algorithm used a numerical minimization method to determine the combination of coefficient values producing the smallest error. The results for all 5 "folds" were combined to determine the best set of coefficients when using that value for λ.

For each of the successive 2000 values of λ in each lasso regression below, the mean and standard error of the associated least squared errors is plotted (red points with grey error bars). As the lasso penalty (λ) is increased, individual regression coefficients become insignificantly different from 0, so that the number of significant coefficients (variables) remaining in the regression becomes smaller. The number of significant coefficients remaining in the regression as λ increases is printed along the top of the plot. The λ at which the best set of coefficients was found (λ_{min}, producing the smallest error) is indicated by the left-hand, vertical dashed line on each plot. The right-hand vertical dashed line identifies the highest value of λ (smallest set of regression variables/coefficients) at which the mean squared error is within one standard error of the minimum. Accepted usage when there are many explanatory variables is that this value of λ ("Lambda min + 1 SEM") identifies the smallest set of regression coefficients that satisfactorily fit the data. These regression coefficients are returned as the result, with "." indicating those coefficients that became zero in the regression.

Supplementary lasso regression 1. Basal OCR dependence on AMPK,TORC1, CGG, genetic group, age and gender.

Lasso regression and variable selection with glmnet:

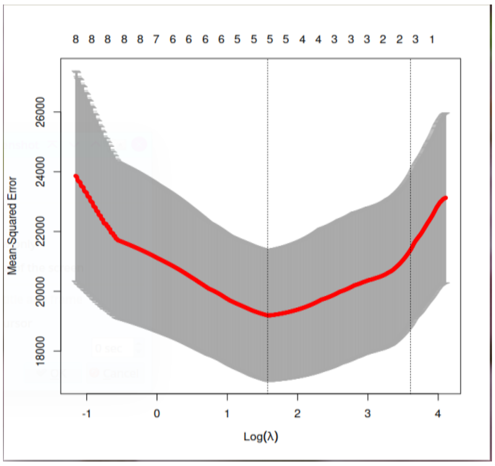


```
[1] "Lambda min + 1 SEM:"

12 x 1 sparse Matrix of class "dgCMatrix"

(Intercept)                    399.60558
Gender.code                     52.74094
Gender.PM                          .
Genetic.Group.code                 .
Log.AMPK                        38.92451
log.AMPK.PM                        .
Log.CGG                            .
Log.CGG.PM                         .
log.Patient.age                    .
log.Patient.age.PM                 .
Log.TORC1.activity                 .
LogTORC1.PM.slope                  .
```

Supplementary lasso regression 2. ATP synthesis OCR dependence on AMPK,TORC1, CGG, genetic group, age and gender.

1. Lasso regression and variable selection with glmnet:

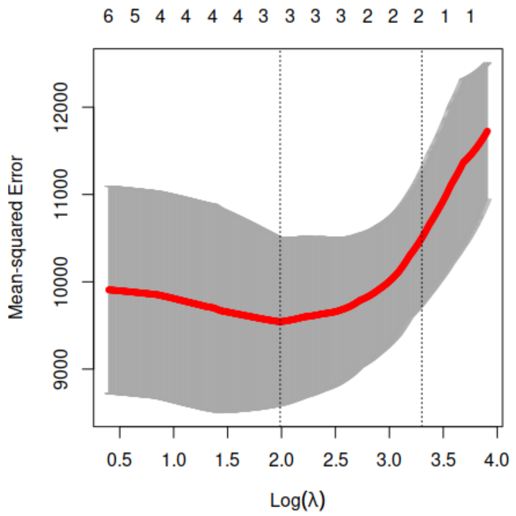


```
[1] "Lambda min + 1 SEM:"

[[4]]
12 x 1 sparse Matrix of class
"dgCMatrix"

(Intercept)                   260.56036
Gender.code                    50.95329
Gender.PM                             .
Genetic.Group.Code                    .
Log.AMPK.                      22.56895
log.AMPK.PM.Slope                     .
Log.CGG.                              .
Log.CGG..PM.slope                     .
log.Patient.age                       .
log.Patient.age.PM                    .
Log.TORC1.activity                    .
LogTORC1.PM.slope                     .
```

Supplementary lasso regression 3. Proton leak dependence on AMPK,TORC1, CGG, genetic group, age and gender

Lasso regression and variable selection with glmnet:

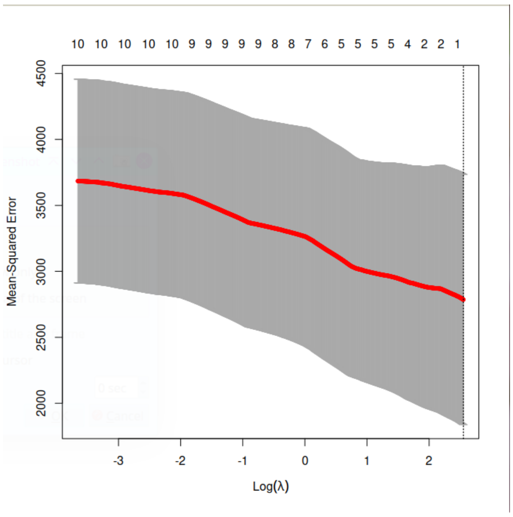


```
[1] "Lambda min + 1 SEM:"

12 x 1 sparse Matrix of class
"dgCMatrix"

(Intercept)              65.13311
Gender.code                     .
Gender.PM                       .
Genetic.Group.code              .
Log.AMPK                        .
Log.AMPK.PM                     .
Log.CGG                         .
Log.CGG.PM                      .
Log.Patient.age                 .
Log.Patient.age.PM              .
Log.TORC1.activity              .
LogTORC1.PM                     .
```

Note: In this regression the smallest mean squared error was found with only the intercept term remaining in the regression ie. inclusion of none of the tested explanatory variables improved the fit to the data.

Supplementary lasso regression 4. Nonmitochondrial OCR dependence on AMPK, CGG, TORC1, genetic group, age and gender.

1. Lasso regression and variable selection with glmnet:

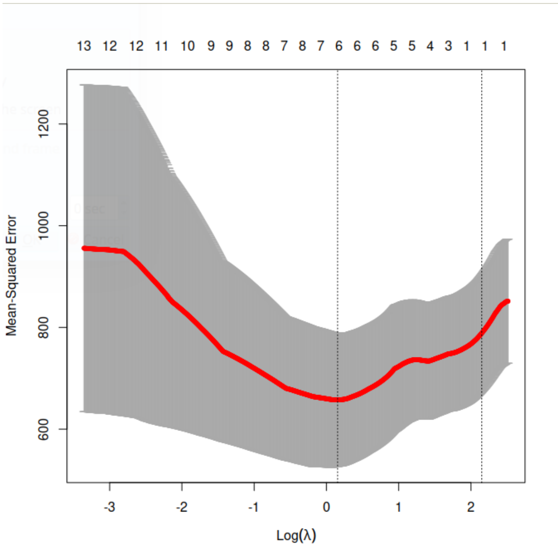


```
[1] "Lambda min + 1 SEM:"

14 x 1 sparse Matrix of class
"dgCMatrix"

(Intercept)                 76.26808
Gender.code                    .
Gender.PM                      .
Genetic.Group.Code             .
Log.AMPK.                      .
log.AMPK.PM                    .
Log.ATP.steady.state.          .
Log.ATPsteady.PM               .
Log.CGG                        .
Log.CGG.PM                     .
Log.Patient.age                .
Log.Patient.age.PM             .
Log.TORC1.activity         -24.66397
LogTORC1.PM                    .
```

Supplementary lasso regression 5. AMPK activity dependence on TORC1, CGG expansion size, ATP steady state levels, genetic group, age and gender.

Lasso regression and variable selection with glmnet:

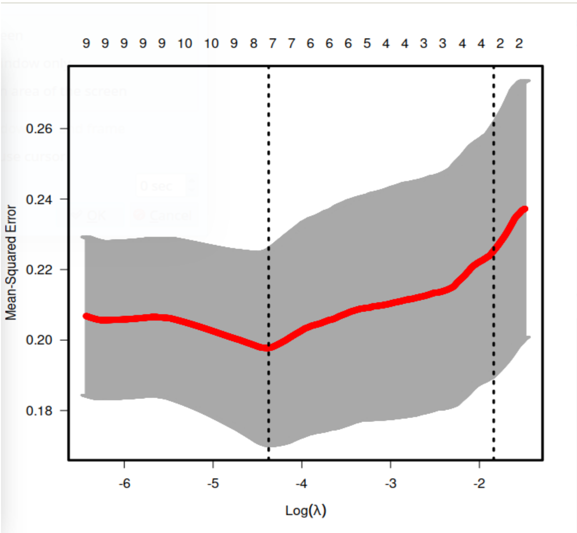


```
[1] "Lambda min + 1 SEM:"

12 x 1 sparse Matrix of class
"dgCMatrix"

(Intercept)                1.1498179
Gender.code                  .
Gender.PM                    .
Genetic.Group.Code         0.0801799
Log.ATP.steady.state         .
Log.ATPsteady.PM             .
Log.CGG                      .
Log.CGG.PM                   .
Log.Patient.age              .
Log.Patient.age.PM           .
Log.TORC1.activity           .
LogTORC1.PM.slope         -0.1713293
```

Supplementary lasso regression 6. TORC1 activity dependence on AMPK, CGG, ATP levels, genetic group, age and gender

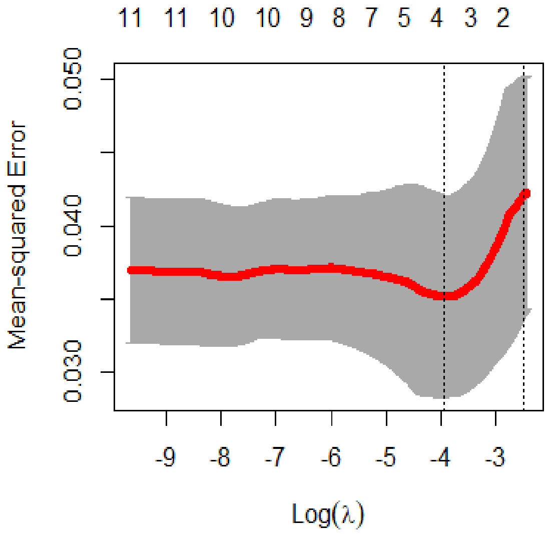


```
[1] "Lambda min + 1 SEM:"

12 x 1 sparse Matrix of class
"dgCMatrix"

(Intercept)                 0.59998770
Gender.code                 .
Gender.PM                   .
Genetic.Group.Code         -0.01002911
Log.AMPK.                   .
log.AMPK.PM.Slope           .
Log.ATP.steady.state.       .
Log.ATPsteady..PM.slope     .
Log.CGG.                    .
Log.CGG..PM.slope           .
log.Patient.age             .
log.Patient.age.PM          .
```

Supplementary lasso regression 7. Lasso multivariate regression of AMPK and TORC1 activities against CGG expansion size.

For this regression, two dependent (Y) variables were used : AMPK and TORC1 activities. The X variables were the genetic group code (allowing the intercepts to differ for the PM group), the log of the CGG expansion size (slope term for the log-linear regression) and a dummy variable allowing the slope of the regression line to differ for the PM and control groups. In addition to the plot of mean squared error against Log(λ), plots of the observed *versus* the predicted Y values, as well as the data points with the calculated best fitting regression lines are shown (*ie*. at the λ value that minimizes the mean square error).

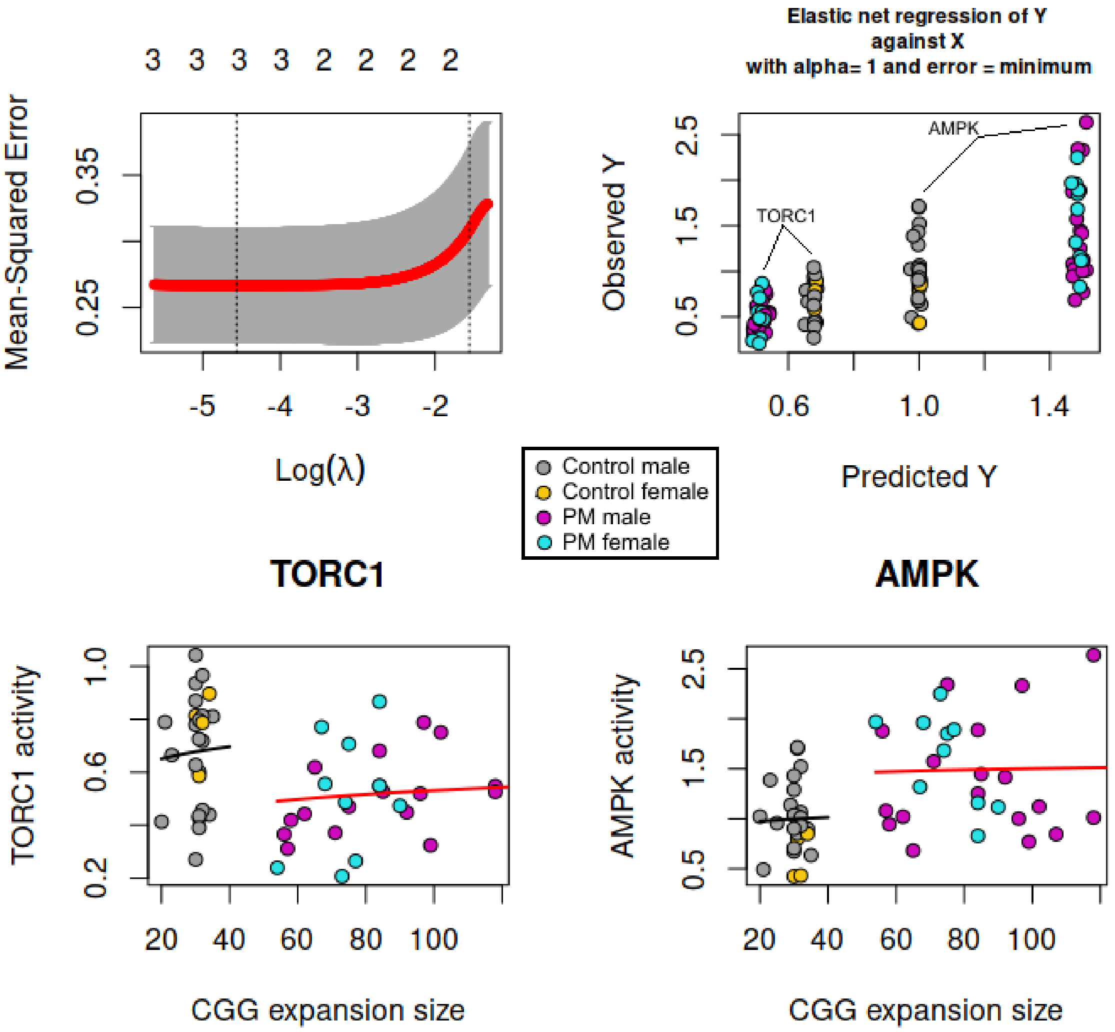


```
Lasso regression and variable selection with glmnet:

$TORC1.activity
4 x 1 sparse Matrix of class "dgCMatrix"
                               1
(Intercept)           0.4544675
Genetic.Group.Code -0.2254402
Log.CGG.              0.1509363
Log.CGG..PM.slope    .

$AMPK.activity
4 x 1 sparse Matrix of class "dgCMatrix"
                               1
(Intercept)           0.8016975
Genetic.Group.Code  0.4352640
Log.CGG.              0.1324907
Log.CGG..PM.slope    .
```

Supplementary lasso regression 8. ATP steady state level dependence on AMPK, TORC1, CGG, ATP synthesis OCR, nonmitochondrial OCR, basal OCR, genetic group, age and gender.

Lasso regression and variable selection with glmnet:

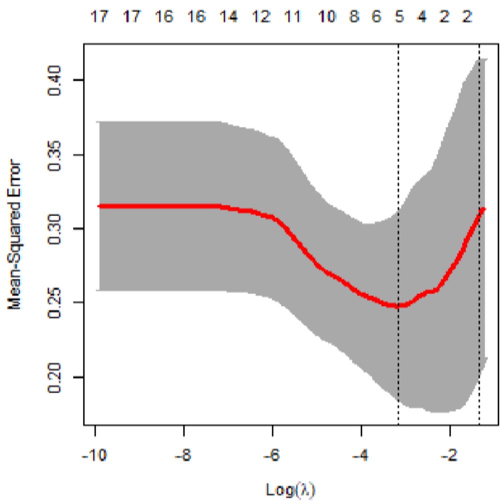


```
[1] "Lambda min + 1 SEM:"
18 x 1 sparse Matrix of class dgCMatrix"

(Intercept)                        1.0895516
Gender.code                         .
Gender.PM                           .
Genetic.Group.code                  .
Log.AMPK.                           .
Log.AMPK.PM.slope                  0.1742841
Log.ATP.average.                    .
Log.ATP.average.PM.slope            .
Log.Basal                           .
Log.Basal.PM                        .
Log.CGG                             .
Log.CGG.PM                          .
Log.Nonrespiratory.average          .
Log.Nonrespiratory.average.PM.slope .
Patient.age                         .
Patient.age.PM                      .
Log.TORC1.activity                  .
LogTORC1.PM                         .
```

Supplementary lasso regression 9. ROS dependence on AMPK, TORC1, CGG, basal OCR, genetic group, age and gender.

Lasso regression and variable selection with glmnet:

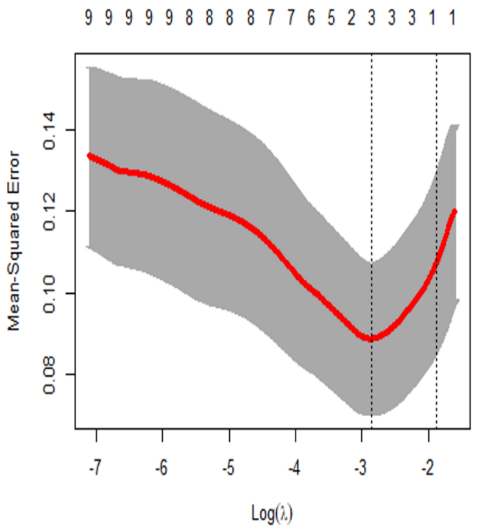


```
[1] "Lambda min + 1 SEM:"
[[4]]
18 x 1 sparse Matrix of class "dgCMatrix"

(Intercept)                  1.03408611
Gender.code                   .
Gender.PM                     .
Genetic.Group.code            .
Log.AMPK                      .
Log.AMPK.PM.slope             .
Log.Basal.                    .
Log.Basal.PM                  .
Log.CGG                       .
Log.CGG.PM                  -0.05821033
Patient.age                   .
Patient.age.PM                .
Log.TORC1.activity            .
Log.TORC1.PM                  .
```
